# Synthesis, Crystal Structures, Antimicrobial Activity, and Acute Toxicity Evaluation of Chiral Zn(II) Schiff Base Complexes

**DOI:** 10.3390/molecules29235555

**Published:** 2024-11-25

**Authors:** Daniela Gutiérrez Arguelles, Claudia P. Villamizar, Eduardo Brambila-Colombres, Bertin Anzaldo, Angel Mendoza, Guadalupe Hernández Téllez, Pankaj Sharma

**Affiliations:** 1 Laboratory Síntesis de Complejos, Faculty Ciencias Químicas, Universidad Autónoma de Puebla, Edif. FCQ-6, C.U. Av. San Claudio y Blvd. 14 Sur, Col. San Manuel, Puebla C.P. 72592, Mexico; 2 Instituto de Química-UNAM, Circuito Exterior, C.U. Coyoacán, Mexico City C.P. 04510, Mexico; 3 Centro de Química del Instituto de Ciencias, Benemérita Universidad Autónoma de Puebla, 18 Sur y Av. San Claudio, Col. San Manuel, Puebla C.P. 72570, Mexico

**Keywords:** chiral imines and zinc complexes, IR and NMR spectroscopic techniques, X-ray crystal structures, antimicrobial activity, acute toxicity

## Abstract

Four mononuclear bioefficient zinc coordination complexes [Zn(NN)_3_](ClO_4_)_2_ (**A**–**D**) involving chiral bidentate Schiff base ligands have been synthesized and characterized by IR, ^1^H, and ^13^C NMR spectroscopy and mass spectrometry. X-ray crystal structures of three of the zinc complexes revealed that the zinc metal ion is hexacoordinated, exhibiting a distorted octahedral geometry where both the nitrogen atoms (NN = pyridyl and imine) of imines are coordinated to the central zinc ion. The isolated zinc complexes were evaluated for their antimicrobial activity in vitro against *Escherichia coli*, *Pseudomonas aeruginosa*, and *Staphylococcus aureus*, displaying varying levels of growth inhibition. An acute toxicity test conducted using *Artemia salina* and Swiss albino mice showed that the zinc complexes **A**–**D** were non-toxic towards *A. salina* at concentrations below 414, 564, 350, and 385 µM, respectively, and did not affect liver biochemical parameters, although pyknosis was induced in hepatocytes of the treated mice.

## 1. Introduction

The design and synthesis of metal–organic complexes have recently gained attention due to their diverse structural possibilities and wide-ranging pharmaceutical applications. These complexes are particularly notable for their biological activities, including antimicrobial, anti-inflammatory, and antitumor effects [1,2]. Transition metals possess unique properties such as charge variation, Lewis acid behavior, and redox activity that allow them to coordinate in three-dimensional ways with different ligands, shaping them to interact with specific molecular targets, thereby facilitating specific molecular interactions [2,3,4].

Schiff bases have been extensively studied for their ease of synthesis, thermal stability, and medicinal properties, such as antitumor, antimicrobial, antiviral, and analgesic activities. The structure of Schiff bases, owing to the fully conjugated π system in their skeletons, contributes to their biological activity [5,6,7]. Both Schiff bases and their transition metal complexes with oxygen and nitrogen donors exhibit significant biological activity, with metal complexes showing enhanced therapeutic potential compared to uncoordinated ligands or free metal ions [8,9,10].

Zinc, the second most abundant trace element in the human body after iron, plays a crucial role in cellular functions. It is present in over 3000 proteins, including those involved in nucleic acid binding, and is essential for enzyme activity, DNA and protein synthesis, and immune function [4,11,12]. Unique properties, such as redox inactivity, flexible coordination geometries, and rapid ligand exchange, make zinc a promising candidate for the development of new anticancer and antibacterial drugs [13,14]. Its ability to prevent free radical reactions further enhances its role in antioxidant defense [15,16,17].

Recent research highlights the potential of Zn(II) complexes, particularly those with N-donor ligands, due to their ease of preparation, reversible electrochemical behavior, and visible light absorption [18,19,20,21]. These complexes have shown significant promise as antitumor agents, with their biological activity influenced by their chemical structure and interactions with cancer cells. Furthermore, exploring optically pure tris-chelate octahedral coordination complexes with asymmetric bidentate ligands offers valuable insights for further drug development [21,22,23].

Zinc(II) complexes also demonstrate broad-spectrum antimicrobial activity, with functionalized pyridine derivatives acting as effective bactericides and fungicides. Divalent zinc ions can work as an effective Lewis acid and can easily adapt to different coordination geometries, in particular, tetrahedral or octahedral [24,25,26,27,28].

In light of the properties of imine compounds and metal complexes, and to expand our ongoing research on chiral imine-based complexes, herein, new chiral imine pyridine ligands and their octahedral enantiomeric pure zinc complexes have been reported. The antimicrobial activity of these Zn(II) complexes was evaluated against *Escherichia coli*, *Pseudomonas aeruginosa*, and *Staphylococcus aureus*. An acute toxicity test of these Zn complexes was also conducted using *Artemia salina* and Swiss albino mice.

## 2. Results and Discussion

### 2.1. Syntheses and Identification by Mass Spectrometry and IR Spectroscopy

**Scheme 1:**
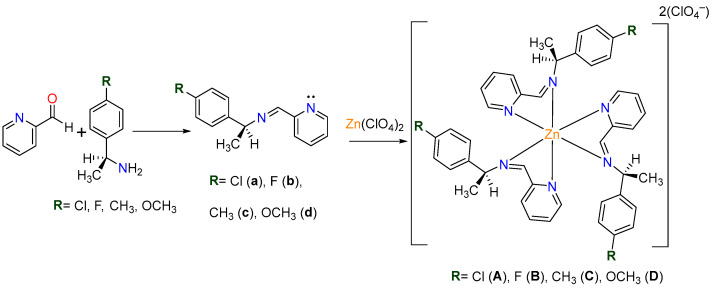
General procedure.

Chiral imines (**a**–**d**) were synthesized under solvent-free conditions via a Schiff base condensation reaction between 2-pyridinecarboxaldehyde and an equimolar amount (1.5 mmol) of primary aryl amines: (*S*)-(-)-1-(4-chlorophenyl)ethylamine, (*R*)-(+)-1-(4-fluorophenyl)ethylamine, (*S*)-(-)-1-(4-methylphenyl)ethylamine, and (*S*)-(-)-1-(4-methoxyphenyl)ethylamine. Coordination of these imine ligands with Zn(ClO_4_)_2_·6H_2_O at ambient temperature achieved the corresponding complexes **A**–**D**, as illustrated in Scheme 1.

The molecular ion peak was observed in the mass spectra in all the ligands, but it was not detected in these four zinc complexes. However, the appearance of the [ZnL_2_ClO₄]^+^ ion peak confirms the molecular formula of the complexes (**A**–**D**). Figures S4, S8, S12 and S16 show the spectra of the imine ligands (**a**, **b**, **c**, and **d**) obtained via positive-ion electron impact (EI) mass spectrometry. Figures S20, S24, S28 and S32 display the spectra for zinc complexes **A**, **B**, **C**, and **D**, respectively, obtained via positive-ion DART mass spectrometry. The observed fragmentation follows a similar pattern for all ligands (**a**–**d**), indicating that the first loss corresponds to a methyl group (M⁺-CH₃), resulting in peaks at 229 *m*/*z* for imine (**a**), 213 *m*/*z* for imine (**b**), 209 *m*/*z* for imine (**c**), and 225 *m*/*z* for imine (**d**). This is followed by the formation of a fragment involving the substituted benzene ring and the chiral carbon, with the loss of the imine group (M⁺-C₆H₅N₂), with peaks at 139 *m*/*z* for imine (**a**), 123 *m*/*z* for imine (**b**), 119 *m*/*z* for imine (**c)**, and 135 *m*/*z* for imine (**d**).

The formation of the Schiff bases (**a**–**d**) is supported by their IR spectra. The absence of significant bands in the 3400–3500 cm^−1^ region confirms the lack of primary amine N–H stretching vibrations. Imine formation is evidenced by strong absorption bands of characteristic ν(C=N) stretching vibrations observed at ~1645 cm^−1^ in these imines.

In the metal complexes (**A**–**D**), the azomethine ν(C=N) stretching vibration shifts to a lower frequency, indicating imine nitrogen coordination to the zinc center. For the zinc complexes, the most intense absorption band around 1080 cm^−1^ is attributed to the vibrations of the perchlorate counterions.

### 2.2. Characterization by NMR Spectroscopy

The chiral imine ligands and their zinc(II) complexes are air-stable, insoluble in water, and soluble in polar organic solvents. In the ^1^H-NMR spectra, the characteristic azomethine proton signal appeared at ~8.45 ppm for the ligand and was shifted to ~8.7 ppm in the complexes, confirming the imine nitrogen coordination to the zinc center.

The methylene group bonded to nitrogen appeared at a lower chemical shift than the free ligands. In the ^13^C-NMR spectra, signals in the 162.68–160.56 ppm range confirmed the deshielding of carbon atom in the C=N (imine) bond, confirming metal–ligand coordination (Table 1).

### 2.3. Single-Crystal X-Ray Analyses of Zinc Complexes

**Figure 1:**
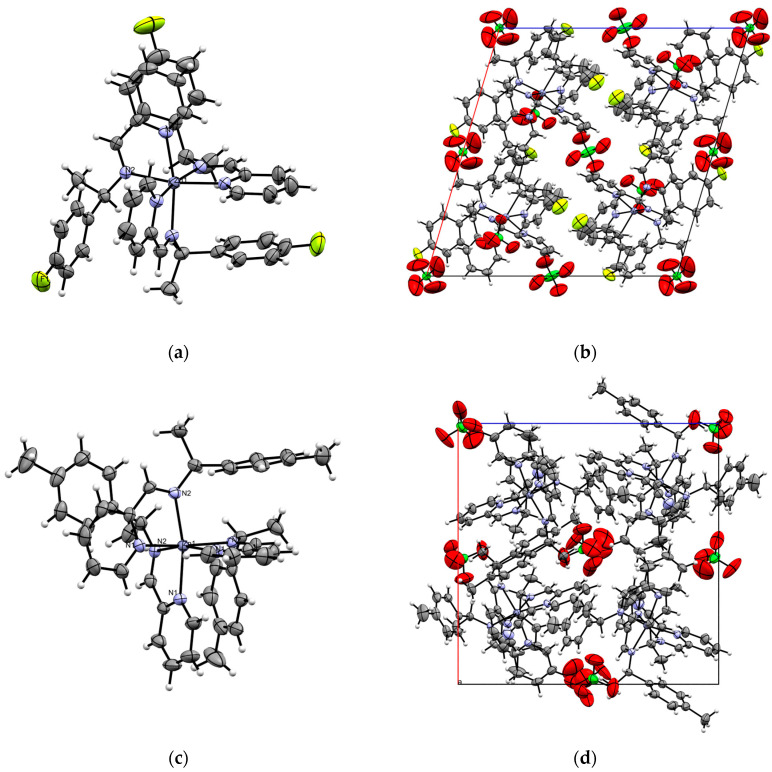

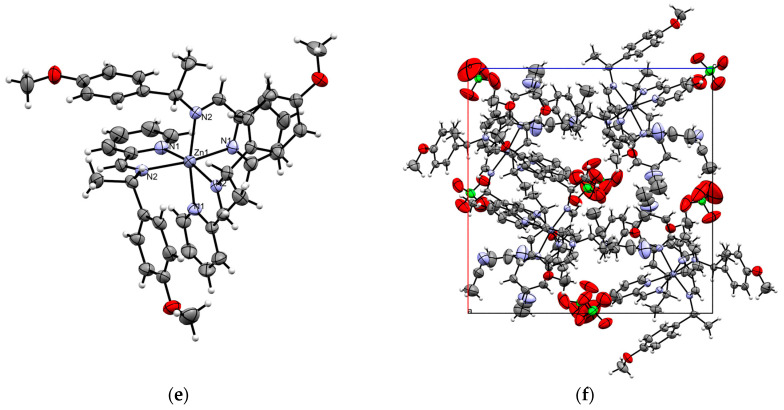
(**a**) ORTEP diagram of complex **B**, with thermal ellipsoids set to a 30% probability level. (**b**) Unit cell along the b-axis for complex **B**. (**c**) ORTEP diagram of complex **C**, with thermal ellipsoids set to 30% probability. (**d**) Unit cell along the b-axis for complex **C**. (**e**) ORTEP diagram of complex **D**, with thermal ellipsoids set to 30% probability. (**f**) Unit cell along the b-axis for complex **D**.

Complexes **C** and **D** crystallize in a cubic crystal system within the P2_1_3 space group, where the asymmetric unit represents one-third of the molecule that is arranged around a threefold rotation axis. In contrast, complex **B** crystallizes in a monoclinic crystal system in the C2 space group, with perchlorate anions present in the unit cell in both cases. The asymmetric units of complexes **B**–**D** are presented in Figure 1 and Table 2.

Single-crystal X-ray diffraction analysis of complexes **B**–**D** reveals that the coordination around the Zn(II) center can be best described as a distorted octahedron, as shown in Figure 1. This distortion is evident from the bond angles, which deviate from the ideal values of 90° for in-plane angles and 180° for axial angles (refer to Table 3). The zinc ion is coordinated by three neutral organic ligands via the pyridine donor and the imine nitrogen atoms, forming three five-membered ring conformations.

Complexes **B**–**D** are facial isomers (*fac*), with all the imine nitrogen atoms positioned on one triangular face of the octahedron. In these zinc complexes, which feature three pyridine and three imine nitrogen atoms, the Zn–N bond lengths are consistent, ranging from 2.16 to 2.2 Å. The coordination trends show that the Zn–N (pyridine) bonds are slightly shorter (2.17 Å) than the Zn–N (imine) bonds (2.2 Å) in complex **B**; while in complexes **C** and **D**, the Zn–N (pyridine) distances are longer as compared to the Zn–N (imine) bonds. The N–Zn–N angle is ~77° in all the complexes, which show slight deviation from a perfect octahedron value. It is to be noted that enantiomeric purity has special relevance in biology, as chirality plays a crucial role in molecular function and interactions in biologically active molecules [29,30]. All the synthesized zinc complexes are optically pure.

Examining the supramolecular properties of complexes **B**–**D** reveals three intramolecular interactions involving parallel-displaced π‧·‧π face-to-face stacking between the pyridine and adjacent phenyl rings (Figure 1b,d,f show the packing diagram of the complexes). The interplanar distances between the centroids of the pyridine and phenyl rings are all less than 4.5 Å, which fall within the expected range for π‧·‧π stacking with horizontal displacement (Table 4). In these Zn complexes, the observed behavior arises largely from steric effects, while the arene π interactions drive the *fac* selectivity. These interactions likely contribute to the stabilization of the complexes in the *fac* configuration, as previously reported [2,3]. The dihedral angle between the planes of the rings is ~14°, indicating a parallel interaction with horizontal displacement. Additionally, these interactions connect neighboring molecules into a 3D network, with perchlorate ions contributing to the supramolecular architecture.

The addition of halogen atoms to organic molecules can significantly alter their properties and enhance biological activity, leading to bulk and conformational changes or increased lipophilicity [14,31]. Empirical studies suggest that incorporating chlorine atom at specific positions can improve its intrinsic biological activity [32]. Likewise, the introduction of a fluorine atom, owing to its high electronegativity, significantly modifies the physicochemical properties of compounds, enhancing pharmacokinetic properties such as metabolic stability and membrane permeability [33].

### 2.4. UV Analysis of Imine Ligands and Zinc Complexes

The UV–Vis spectra of all compounds were recorded in ACN, as illustrated in Figures S5, S10, S15, S20, S25, S30, S35, and S40. The imine ligands (**a**–**d**) exhibit two main characteristic absorption bands: a strong absorption peak at ~271 nm, corresponding to π–π* transitions in the aromatic systems. Among these, the OCH_3_ group (imine **a**) induces the most significant bathochromic shift and extended conjugation, followed by CH_3_ (imine **c**), Cl (imine **a**), and F (imine **b**). This tendency is a result of the ability of OCH_3_ to stabilize the conjugated system through both resonance and inductive effects. Additionally, a high-intensity peak appears around ∼200 nm, which is attributed to the n–π * transition.

In complexes **A**–**D**, the π–π* transition band exhibits a red shift in the maximum absorption wavelength, attributed to the increased electron density of the unsaturated system upon coordination with the zinc ion. No *d–d* transitions are observed in these complexes. This slight difference in the absorbance between the imine ligands (**a**–**d**) and their respective complexes (**A**–**D**) suggests that the absorption is more strongly influenced by the extent of conjugation within the system.

### 2.5. Antimicrobial Activity

The antimicrobial activity results are summarized in Table 5, showing that the MIC values for compounds **A**–**C** are higher against *Escherichia coli* (Gram-negative) and *Pseudomonas aeruginosa* (Gram-negative), indicating that larger doses are required to inhibit their growth compared to *Staphylococcus aureus* (Gram-positive). These findings indicate that the complexes were more effective against Gram-positive bacteria than Gram-negative bacteria. This difference suggests that the antimicrobial properties of metal complexes are influenced by the structural composition of the bacterial cell wall, including the cytoplasmic membrane, the peptidoglycan layer, and the lipopolysaccharide-containing outer membrane, which can act as a barrier to the entry of the complexes [34,35,36].

Zinc(II) complexes exhibit greater antimicrobial activity than their Schiff base ligands, attributed to their lipophilic nature, which facilitates membrane crossing [37,38]. Chelation plays a critical role by reducing the polarity of metal ions through orbital interactions, redistributing the positive charge between the metal ion and donor atoms. The delocalization of π-electrons across the chelate ring, further increasing the lipophilicity of the metal complexes and improving their interaction with biological membranes. The results also revealed that the antimicrobial activity of the complexes was significantly influenced by their geometries [39,40,41,42].

The brine shrimp assay is a useful method for predicting compound toxicity, offering an efficient, rapid, and inexpensive test with a strong correlation to cytotoxic activity. The median lethal concentrations (LC_50_) from the brine shrimp lethality assay are presented in Table 6. The data can be extrapolated to estimate LC_50_ values in other biological models [43]. For example, Largarto [44] reported a good correlation (r = 0.85; *p* < 0.05) between the LC_50_ from the brine shrimp lethality assay and the LD_50_ from acute oral toxicity assays in mice. All tested compounds exhibited biological activity in this model, with complex **A** showing the highest lethality in brine shrimp (LC_50_ = 414 µM). These results were used as a guide for subsequent tests in mice.

### 2.6. Acute Toxicity Test

**Figure 2:**
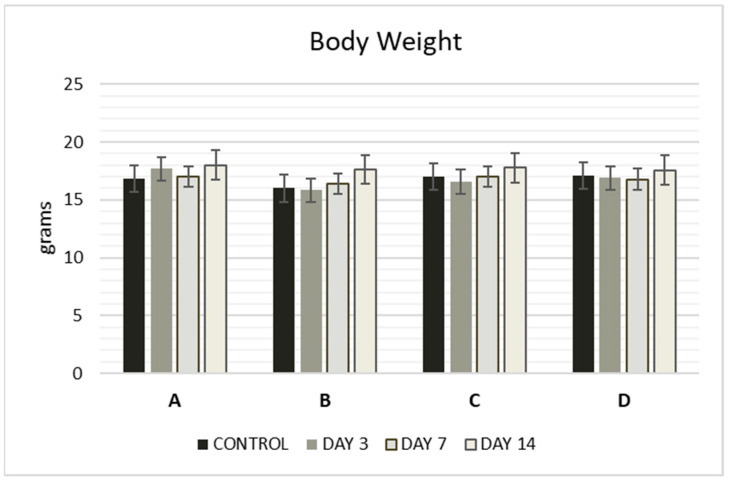
Body weight (g) of mice at 3, 7, and 14 days after administration of complexes **A**–**D**. The compounds were administered intraperitoneally (i.p.) as a single dose. Values are expressed as mean ± S.E.M. of 3 animals. Significant differences compared to the control group, *p* < 0.05. The control group was administered 10% DMSO and saline.

The acute toxicity of the Zn complexes was evaluated at three concentrations: 500, 100, and 10 mg/kg. Mortality was recorded over 48 h to calculate the median lethal dose (LD_50_), as shown in Table 7. LD_50_ values for complexes **A**–**D** were determined using Lorke’s method (Table 6), with complex **A** being the most lethal, exhibiting the highest value (500 mg/kg), nearly double that of the other compounds. No significant changes in relative body weight were observed in the mice after administration, and no significant differences (*p* < 0.05) were detected when compared to the control group (Figure 2).

**Figure 3:**
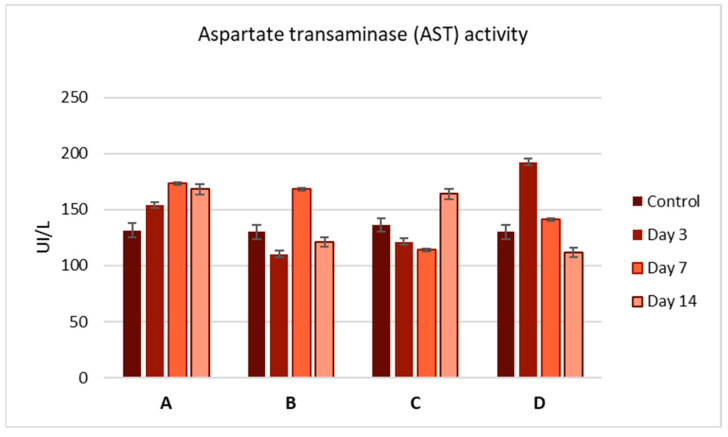
Aspartate aminotransferase (AST) activity (U/L) in the serum of mice at 3, 7, and 14 days after a single-dose administration of complexes **A**–**D**. Values are expressed as mean ± S.E.M. for 3 animals. *p* < 0.05.

**Figure 4:**
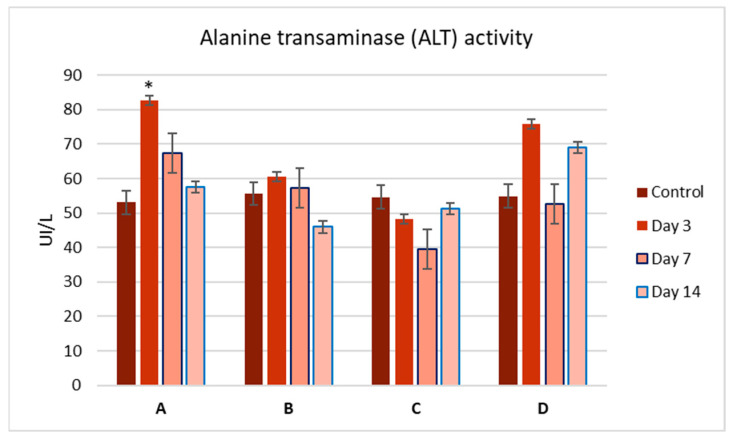
Alanine aminotransferase (ALT) activity (U/L) in the serum of mice at 3, 7, and 14 days after a single-dose administration of complexes **A**–**D**. Values are expressed as mean ± S.E.M. for 3 animals. *p* < 0.05.

**Figure 5:**
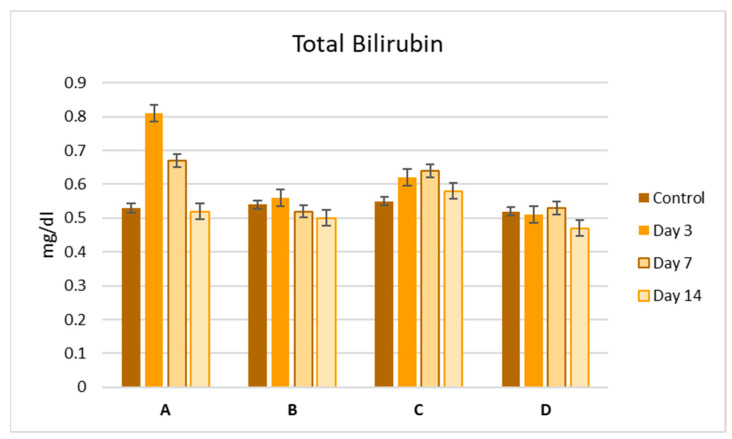
Bilirubin levels (mg/dL) in the serum of mice at 3, 7, and 14 days after a single-dose administration of complexes **A**–**D**. Values are expressed as mean ± S.E.M. for 3 animals. *p* < 0.05.

To evaluate the side effects of the Zn complexes, we performed liver function tests by analyzing serum samples for AST, ALT, and bilirubin levels (Figure 3, Figure 4 and Figure 5). No significant differences were observed between the control and treatment groups, except for complex **C**, which showed a slight increase in ALT activity on day 3 (Figure 4). Overall, the biochemical parameters suggest that complexes **A**–**D** do not impair liver function, as no significant changes were noted post-administration.

Histological analysis of the liver revealed that hepatocytes in the control group retained normal morphology, with uniform parenchyma and no signs of necrosis. In contrast, hepatocytes from the treated groups exhibited pyknosis, characterized by chromatin condensation—indicative of both apoptotic and necrotic cell death. Despite the observed pyknosis, the absence of membrane disruption and minimal changes in liver enzyme levels imply that complexes **A**–**D** induce apoptosis rather than necrosis in the liver cells of treated mice.

## 3. Materials and Methods

^1^H-NMR and ^13^C-NMR spectra were recorded on a JEOL 400S spectrometer (JEOL Ltd., Tokyo, Japan). Spectra of the ligands were recorded in CDCl_3_, while those of the zinc complexes were recorded in (CD_3_)_2_CO. Electron Ionization (EI) and Direct Analysis in Real Time (DART) mass spectrometry spectra were acquired on a JEOL JMS-AX505 HA mass spectrometer (JEOL Ltd., Tokyo, Japan), operated in positive-ion mode. IR spectra were recorded on a Nicolet Magna 750 FT-IR spectrophotometer using KBr disks (SpectraLab Scientific Inc., Markham, ON, USA). Melting points were measured using a Melt-Temp II apparatus and are uncorrected (American Laboratory Trading, San Diego, CA, USA). All reagents were obtained from commercial sources and used as received. The optical rotation was obtained on a Perkin Elmer 241 polarimeter (PerkinElmer, Waltham, MA, USA). The UV–Vis absorption spectra were obtained in a range of 200–500 nm using a Shimadzu UV–Vis 160 spectrophotometer (Shimadzu, Tokyo, Japan). Solvents were purified by standard methods and freshly distilled prior to use.

### 3.1. Synthesis of Imines (***a***–***d***) and Complexes (***A***–***D***)

Chiral imines (**a**–**d**) were synthesized under solvent-free conditions via a Schiff base condensation reaction between 2-pyridinecarboxaldehyde and an equimolar amount (1.5 mmol) of primary aryl amines: (*S*)-(-)-1-(4-chlorophenyl)ethylamine, (*R*)-(+)-1-(4-fluorophenyl)ethylamine, (*S*)-(-)-1-(4-methylphenyl)ethylamine, and (*S*)-(-)-1-(4-methoxyphenyl)ethylamine. The reaction of these imine ligands with Zn(ClO_4_)_2_·6H_2_O at ambient temperature led to the formation of the corresponding complexes **A**–**D**.

**Imine a:** Yield: 90%, color: Ligt yellow oil, FT-IR (ν, cm^−1^): 2979 ν_as_(C-H); 1647 ν_s_(C=N), 824 ν_δ_(C-H), 772 cm^−1^ ν_as_(C-Cl). ^1^H-NMR (400 MHz, CDCl_3_/TMS). 1.57 (*d*, ^3^J = 8 Hz, 3H, H-3), 4.60 (*q*, ^3^J = 4 Hz, 1H, H-2), 7.29 (*m*, 1H, H12), 7.32 (*m*, 2H, H-5, H-9), 7.38 (*m*, *2*H, H-6, H-8), 7.74 (*m*, 1H, H-14), 8.09 (*m*, 1H, H-13), 8.45 (*s*, 1H, H-10), 8.63 (*m*, 1H, H-15); ^13^C-NMR (100 MHz, CDCl_3_/TMS, δ: ppm): 24.09 (C-3), 68.90 (C-2), 121.50 (C-12), 124.85 (C-14), 128.08 (C-5, C-9), 128.61 (C-6, C-8), 132.67 (C-7), 136.58 (C-13), 143.12 (C-4), 149.44 (C-15), 154.60 (C-11 H*C*=N), 160.70 (C-10). MS–EI: *m*/*z* = 244.72 (M^+^) C_14_H_13_ClN_2_; 229 (M^+^-C_13_H_10_ClN_2_); 239 (M^+^-C_8_H_8_Cl); ${\left[\alpha \right]}_{D}^{20}\;$= +30.8° (c 1, CHCl_3_). UV–Vis(ACN): λ (nm) (ε (mol^−1^ dm^3^ cm^−1^)) 271 (4736).

**Imine b:** Yield: 94%, color: ligth yellow oil; FT-IR (ν, cm^−1^): 3055 ν_as_(C-H_Ar_); 2973 ν_as_(C-H), 1646 cm^−1^ ν_s_(C=N), 1436 cm^−1^ ν_as_ (C-F), 833 ν_δ_(C-H); ^1^H-NMR (400 MHz, CDCl_3_/TMS, δ = ppm): 1.59 (*d*, ^3^J = 8 Hz, 3H, H-3), 4.63 (*q*, ^3^J = 4 Hz, 1H, H-2), 7.08 (*m*, 2H, H-5, H-9), 7.31 (*m*, 1H, H-12), 7.40 (*m*, *2*H, H-6, H-8), 7.74 (*m*, 1H, H-14), 8.08 (*m*, 1H, H-13), 8.45 (*s*, 1H, H-10), 8.63 (*m*, 1H, H-15); ^13^C-NMR (100 MHz, CDCl_3_/TMS, δ: ppm): 24.73 (C-3), 68.88 (C-2), 115.17 (C-6, C-8), 121.50 (C-12), 124.82 (C-14), 128.18 (C-5, C-9), 136.57 (C-13), 140.30 (C-4), 149.92 (C-15), 154.64 (C-11), 160.56 (C-10), 162.84, 161.3 (C-7). MS–EI: *m*/*z* = 228.26(M^+^)C_14_H_13_FN_2_; 213 (M^+^-C_13_H_10_FN_2_); 123 (M^+^-C_8_H_8_F); ${\left[\alpha \right]}_{D}^{20}\;$= −34.8° (c 1, CHCl_3_). UV–Vis(ACN): λ (nm) (ε (mol^−1^ dm^3^ cm^−1^)) 270 (3666).

**Imine c:** Yield: 82%, color: yellow oil, FT-IR (ν, cm^−1^): 2971 ν_as_(C-H); 1645 cm^−1^ ν_s_(C=N), 816 ν_δ_(C-H); ^1^H-NMR (400 MHz, CDCl_3_/TMS, δ = ppm): 1.60 (*d*, ^3^J = 8 Hz, 3H, H-3), 2.33 (*s*, 3-H, H-16), 4.61 (*q*, ^3^J = 4 Hz, 1H, H-2), 7.17 (*d*, ^3^J = 7.5 Hz 2H, H-6, H-8), 7.30 (*m*, 1H, H-15), 7.33 (*d*, ^3^J = 8 Hz *2*H, H-5, H-9), 7.73 (*t*, ^3^J = 8 Hz 1H, H-14), 8.08 (*d*, ^3^J = 8 Hz, 1H, H-12), 8.45 (*s*, 1H, H-10), 8.63 (*m*, 1H, H-15); ^13^C-NMR (100 MHz, CDCl_3_/TMS, δ: ppm): 21.10 (C-10), 24.46 (C-3), 69.34 (C-2), 121.49 (C-12), 124.68 (C-14), 126.65 (C-5, C-9), 129.20 (C-6, C-8), 136.50 (C-13), 136.64 (C-7), 141.55 (C-4), 149.35 (C-15), 154.84 (C-11), 160.30 (C-10). MS–EI: *m*/*z* = 224.30 (M^+^) C_15_H_16_N_2_; 209 (M^+^-C_14_H_13_N_2_); 119 (M^+^-C_9_H_11_);$\;{\left[\alpha \right]}_{D}^{20}$ = +28.3° (c 1, CHCl_3_). UV–Vis(ACN): λ (nm) (ε (mol^−1^ dm^3^ cm^−1^)) 274 (22,827).

**Imine d:** Yield: 79%, color: yellow oil, FT-IR (ν, cm^−1^): 2969 ν_as_(C-H), 1644 cm^−1^ ν_s_(C=N), 1034 ν_s_(COC), 830 ν_δ_(C-H); ^1^H-NMR (400 MHz, CDCl_3_/TMS, δ = ppm): 1.36 (*d*, ^3^J = 8 Hz 3H, H-3), 3.79 (*s*, 3-H, H-16), 4.61 (*q*, ^3^J = 4 Hz, 1H, H-2), 6.89 (*d*, ^3^J = 4 Hz, 2H, H-6, H-8), 7.25 (*m*, 1H, H-14), 7.36 (*d*, ^3^J = 8 Hz, *2*H, H-5, H-9), 7.71 (*m*, 1H, H-13), 8.08 (*d*, ^3^J = 4 Hz, 1H, H-12), 8.44 (*s*, 1H, H-10), 8.61 (*m*, 1H, H-15); ^13^C-NMR (100 MHz, CDCl_3_/TMS, δ: ppm): 24.40 (C-3), 55.30 (C-16), 68.96 (C-2), 113.88 (C-5, C-9), 121.49 (C-12), 124.69 (C-14), 127.80 (C-5, C-9), 136.52 (C-13), 136.00 (C-4), 149.35 (C-15), 154.81 (C-11), 158.62 (C-7), 160.19 (C-10). MS–EI: *m*/*z* = 240.30 (M^+^) C_15_H_16_N_2_O; 225 (M^+^-C_14_H_13_N_2_O); 135 (M^+^-C_9_H_11_O); ${\left[\alpha \right]}_{D}^{20}$ = +16.8° (c 1, CHCl_3_). UV–Vis(ACN): λ (nm) (ε (mol^−1^ dm^3^ cm^−1^)) 273 (10,312).

**Complex A:** Yield: 94%, color: pale yellow-white, Mp 154 °C. FT-IR (ν, cm^−1^): 2982 ν_as_(C-H), 1597 ν_s_(C=N), 828 ν_δ_(C-H), 786 ν_s_(C-Cl), ^1^H-NMR (400 MHz, C_3_D_6_O/TMS, δ = ppm): 1.73 (*d*, ^3^J = 4 Mz, 3H, H-3), 5.89 (*q*, ^3^J = 4 MHz, 1H, H-2), 6.91 (*d*, ^3^J = 8 MHz,, 2H, H-6, H-8), 6.96 (*d*, ^3^J = 8 MHz, *2*H, H-5, H-9), 7.45 (*m*, 3H, H-12, H-13, H-14), 8.29 (*m*, 1H, H-15), 8.74 (*s*, 1H, H-10). ^13^C-NMR (100 MHz, C_3_D_6_O/TMS, δ: ppm): 22.89 (C-3), 63.52 (C-2), 127.75 (C-6, C-8), 128.66 (C-5, C-9), 129.84 (C-7), 129.99 (C-4), 132.79 (C-12), 139.82 (C-14), 141.29 (C-13), 146.49 (C-11), 148.22 (C-15), 162.68 (C-10). MS–DART: *m*/*z* = 655 [ML_2_ClO_4_]^+^, 544 [C_28_H_26_Cl_2_N_4_O_4_Zn]^+^; ${\;\left[\alpha \right]}_{D}^{20}\;$= −281.6° (c 1, CHCl_3_). UV–Vis(ACN): λ (nm) (ε (mol^−1^ dm^3^ cm^−1^)) 283 (10,312).

**Complex B:** Yield: 90%, color: pale yellow-white, Mp 179 °C. FT-IR (ν, cm^−1^): 2987 ν_as_(C-H), 1597 cm^−1^ (C=N), 1015 (C-F), 845 ν_δ_(C-H); ^1^H-NMR (400 MHz, C_3_D_6_O/TMS, δ = ppm): 1.73 (*d*, ^3^J = 4 MHz, 3H, H-3), 5.89 (*q*, ^3^J = 4 MHz, 1H, H-2), 6.68 (*m*, 2H, H-5, H-9), 6.93 (*m*, 2H, H-6, H-8), 7.68 (*m*, 3H, H-12, H-13), 7.85 (*m*, 1H, H-14), 7.29 (*m*, 1H, H-15), 8.71 (*s*, 1H, H-10). ^13^C-NMR (100 MHz, C_3_D_6_O/TMS, δ: ppm): 23.14 (C-3), 63.51 (C-2), 115.28, 115.46 (C-6, C-8), 127.87, 127.93 (C-5, C-9), 129.76 (C-12), 129.93 (C-14), 141.52 (C-13), 146.63 (C-15), 148.10 (C-11), 162.40 (C-10). MS–DART: *m*/*z* = 621 [ML_2_ClO_4_]^+^, 520 [C_28_H_26_F_2_N_4_O_4_Zn]^+^; ${\left[\alpha \right]}_{D}^{20}\;$= +331.4° (c 1, CHCl_3_). UV–Vis(ACN): λ (nm) (ε (mol^−1^ dm^3^ cm^−1^)) 273 (2463.5).

**Complex C:** Yield: 88%, color: pale yellow-white, Mp 154 °C. FT-IR(ν, cm^−1^): 2980 ν_as_(C-H), 1597 ν_s_(C=N), 822 ν_δ_(C-H); ^1^H-NMR (400 MHz, C_3_D_6_O/TMS, δ = ppm): 1.66 (*d*, ^3^J = 8 MHz, 3H, H-3), 2.22 (*s*, 3-H, H-16), 5.43 (*q*, ^3^J = 4 MHz, 1H, H-2), 6.54 (*d*, ^3^J = 8 MHz, 2H, H-6, H-8), 6.70 (*d*, ^3^J = 8 MHz, *2*H, H-5, H-9), 7.41 (*m*, 1H, H-14), 7.47 (*m*, 1H, H-13), 7.54 (*m*, 1H, H-12), 8.11 (*m*, 1H, H-15), 8.23 (*s*, 1H, H-10). ^13^C-NMR (100 MHz, C_3_D_6_O/TMS, δ: ppm): 20.16 (C-3), 22.80 (C-16), 64.17 (C-2), 125.86 (C-6, C-8), 129.47 (C-5, C-9), 129.51 (C-12), 129.90 (C-7), 129.90 (C-4), 137.61 (C-14), 141.26 (C-13), 146.49 (C-11), 147.98 (C-15), 161.55 (C-10). MS–DART: *m*/*z* = 611 [ML_2_ClO_4_]^+^, 512 [C30H32N_4_Zn]^+^; ${\;\left[\alpha \right]}_{D}^{20}\;$= −306.4° (c 1, CHCl_3_) UV–Vis(ACN): λ (nm) (ε (mol^−1^ dm^3^ cm^−1^)) 274 (8422.9).

**Complex D:** Yield:79%, color: pale yellow-white, Mp 152 °C; FT-IR (ν, cm^−1^): 2980 ν_as_(C-H), 1597 ν_s_(C=N), 1245 ν_s_(COC), 822 ν_δ_(C-H); ^1^H-NMR (400 MHz, C_3_D_6_O/TMS, δ = ppm): 1.64 (*d*, ^3^J = 8 MHz 3H, H-3), 3.75 (*s*, 3-H, H-16), 5.41 (*q*, ^3^J = 4 MHz, 1H, H-2), 6.45 (*d*, ^3^J = 8 MHz, 2H, H-6, H-8), 6.57 (*d*, ^3^J = 8 MHz, *2*H, H-5, H-9), 7.39 (*m*, 1H, H-13), 7.54 (*m*, 2H, H-12, H-14), 8.10 (*m*, 1H, H-15), 8.30 (*s*, 1H, H-10). ^13^C-NMR (100 MHz, CD_3_CN/TMS, δ: ppm): 23.23 (C-3), 55.53 (C-16), 64.22 (C-2), 114.50 (C-6, C-8), 127.48 (C-5, C-9), 129.84 (C-12), 130.14 (C-4), 132.74 (C-14), 141.81 (C-13), 146,91 (C-11), 148.27 (C-15), 15.37 (C-7),161.77 (C-10). MS–DART: *m*/*z* = 643 [ML_2_ClO_4_]^+^, 544 [C_30_H_32_N_4_O_2_Zn]^+^; ${\;\left[\alpha \right]}_{D}^{20}\;$= −230.1° (c 1, CHCl_3_); UV–Vis(ACN): λ (nm) (ε (mol^−1^ dm^3^ cm^−1^)) 275 (9618.8).

### 3.2. Crystallographic Study

Single crystals for X-ray structure determination of complexes **B**, **C,** and **D** were obtained by slow evaporation of CH_2_Cl_2_ solutions and manipulated in ambient air. A summary of the crystallographic results is presented in Table 2. Diffraction data were collected at 293 K on a Bruker P4 diffractometer, using graphite-monochromatized Mo Kα radiation (λ = 0.71073 Å), following standard procedures [45]. Absorption effects were corrected using scans’ data [46], and structures were refined using the SHELX suite of programs [47].

### 3.3. Antimicrobial Studies

Complexes **A**–**D** were screened for their in vitro antibacterial activity against two Gram-negative bacterial strains, *Escherichia coli* (ATCC 25922) and *Pseudomonas aeruginosa* (ATCC 27853), and one Gram-positive bacterial strain, *Staphylococcus aureus* (ATCC 25923). The antibacterial activities of all compounds were evaluated by determining their minimal inhibitory concentrations (MICs) using the broth microdilution method. Bacterial strains were reconstituted in sterile distilled water, cultured in Mueller–Hinton broth (MHB), and incubated at 37 °C for 24 h. Compound dilutions were prepared in DMSO, with concentrations ranging from 25 µg/mL to 0.48 µg/mL. The negative control consisted of MHB without bacteria, the positive control consisted of MHB with bacteria only, and penicillin was used as a reference antibiotic. DMSO was also tested and showed no antibacterial activity against any of these strains.

#### Brine Shrimp Assay

*Artemia salina* eggs were hatched in artificial seawater prepared with 34 g/L of commercial sea salt. After 24 h, the shrimp matured as nauplii and were ready for the assay. For each complex, four concentrations (tested in triplicate) were evaluated to establish a dose–response relationship, with a control group using DMSO as the vehicle for dilutions. The tested concentrations were 1000, 500, 250, and 125 µM. Each well contained 10 brine shrimp larvae, and the total volume was adjusted to 100 µL with artificial salt water. After 24 h, live larvae were counted, and the LC_50_ value was estimated using statistical methods.

### 3.4. Acute Toxicity Test

For the determination of acute toxicity, 150 female Swiss albino mice (8 weeks old) were obtained from BUAP. The animals were housed in an animal room maintained at 22 ± 3 °C with a 12 h light/dark cycle. They were divided into two control groups and 48 treated groups, each consisting of three animals. Three single doses (10, 100, and 500 mg/kg) were tested to establish a dose–response relationship, and the animals were monitored for 14 days. Solutions were prepared in 10% DMSO as the vehicle and were administered via intraperitoneal injection. One control group received saline only, while the other received saline with 10% DMSO. The test followed OECD standards for acute toxicity studies. Mortality was recorded during the first 48 h to calculate the LD_50_ according to Lorke’s method [48].

#### 3.4.1. Body Weight and Effects on Liver Function

The animals treated with zinc complexes were weighed on days 3, 7, and 14 during the two-week period. Aspartate aminotransferase (AST), alanine aminotransferase (ALT), and bilirubin levels were determined from serum samples to evaluate liver function and possible hepatic injury. Only the animals that survived the 14-day period were euthanized using pentobarbital sodium; their livers were removed for macroscopic analysis and preserved in 4% buffered formalin solution for histological study.

#### 3.4.2. Histopathology Analysis

All samples were preserved in 4% buffered formaldehyde, embedded in paraffin, and sectioned at a thickness of five microns using a Microm HM310 rotary microtome (New Life Scientific, Cridersville, OH, USA). The sections were stained with hematoxylin and eosin and examined under a Leica light microscope (Leica Microsystems, Tokyo, Japan).

#### 3.4.3. Statistical Analysis

Results are expressed as mean ± standard error of the mean (S.E.M). Comparisons between groups were performed using the ANOVA test. A probability level of *p* < 0.05 was considered statistically significant.

## 4. Conclusions

Optically pure pyridyl imine ligands were synthesized by the direct reaction of 2-pyridinecarboxaldehyde with an equimolar amount of optically active primary amine, followed by coordination with a Zn metal precursor. Three imine ligands coordinated through pyridinyl and imine nitrogen atoms, resulting in octahedral coordination around the zinc center. All the compounds were characterized by IR, ^1^H, and ^13^C NMR spectroscopy and mass spectrometry.

In the ^1^H-NMR spectra, the downfield shifts observed for the azomethine protons and aromatic hydrogens suggest coordination to the metal center, which is further supported by the IR spectra showing shifts in the azomethine (-HC=N-) bands when compared with the synthesized imine ligands. The octahedral geometry around the Zn(II) center exhibits minimal distortion, with the optically pure chiral precursor enforcing a *fac* configuration. Additionally, π–π interactions contribute to the stabilization of the supramolecular structure, as consistently observed in the crystal lattices.

The synthesized metal complexes were evaluated for their biological activity, including acute toxicity in brine shrimp and mice models. These complexes demonstrated promising antimicrobial activity, particularly against Gram-negative bacteria, while exhibiting low cytotoxicity in both *A. salina* and mice models.

## Figures and Tables

**Table 1:** Chemical shifts and IR bands of imine ligands (**a**–**d**) and their complexes (**A**–**D**).

Compound	FT-IR (C=N) (cm^−1^)	NMR ^1^H (ppm)	NMR ^13^C (ppm)
Imina [**a**]	1647	1.57 (H-3), 4.60 (H-2), 8.45 (H-10)	24.09 (C-3), 68.90 (C-2), 160.70 (C-10)
Imina [**b**]	1646	1.59 (H-3), 4.63 (H-2), 8.45(H-10)	24.73(C-3), 68.88(C-2), 160.56 (C-10)
Imina [**c**]	1645	1.63 (H-3), 4.61 (H-2), 8.45 (H-10)	24.46 (C-3), 69.34 (C-2), 160.30 (C-10)
Imina [**d**]	1644	1.36 (H-3), 4.61 (H-2), 8.44 (H-10)	24.40 (C-3), 68.96 (C-2), 160.19 (C-10)
Complex **A**	1597	1.73 (H-3), 5.89 (H-2), 8.44 (H-10)	22.89 (C-3), 63.52 (C-2), 162.68 (C-10)
Complex **B**	1597	1.73 (H-3), 5.89 (H-2), 8.71 (H-10)	23.14 (C-3), 63.51 (C-2), 162.40 (C-10)
Complex **C**	1597	1.66 (H-3), 5.43 (H-2), 8.23 (H-10)	20.16 (C-3), 64.17 (C-2), 161.55 (C-10)
Complex **D**	1597	1.64 (H-3), 5.41 (H-2), 8.30 (H-10)	23.23 (C-3), 64.22 (C-2), 161.77 (C-10)

**Table 2:** Crystal data and refinement parameters for zinc complexes **B**, **C**, and **D**.

Compound	Complex B	Complex C	Complex D
Chemical formula	C_42_H_39_Cl_2_F_3_N_6_O_8_Zn	C_47_H_51_Cl_2_N_7_O_8_Zn	C_51_H_57_Cl_2_N_9_O_11_Zn
Molar mass (g mol^−1^)	949.06	978.24	1108.35
*T* (K)	293(2)	293(2)	293(2)
Crystal system	Monoclinic	Cubic	Cubic
Space group	*C*2	*P*2_1_3	*P*2_1_3
*a* (Å)	19.2189(9)	16.9023(3)	17.5430(3)
*b* (Å)	12.9740(5)	16.9023(3)	17.5430(3)
*c* (Å)	18.6227(11)	16.9023(3)	17.5430(3)
*α* (°)	90	90	90
*β* (°)	106.389(6)	90	90
*γ* (°)	90	90	90
*V* (Å^3^)	4454.8(4)	4828.8(3)	5399.0(3)
*Z*	4	4	4
*p*_calc_ (g cm^−3^)	1.415	1.346	1.364
*μ* (mm^−1^)	0.741	0.678	0.620
*F*(000)	1952.0	2040.0	2312.0
Crystal size (mm^3^)	0.297 × 0.283 × 0.164	0.485 × 0.287 × 0.231	0.64 × 0.504 × 0.381
Radiation (*λ*/Å)	MoK*α* (*λ* = 0.71073)	MoK*α* (*λ* = 0.71073)	MoK*α* (*λ* = 0.71073)
2Θ range (°)	6.28–55.746	5.904–54.17	6.57–52.694
Reflections collected	30,747	17,438	18,653
*R*_int_	0.0338	0.0308	0.0351
GOF on *F*^2^	1.027	1.049	1.070
*R*_1_, *wR*_2_ (*I* ≥ 2*σ* (I))	0.0463, 0.1183	0.0367, 0.0952	0.0461, 0.1205
*R*_1_, *wR*_2_ (all data)	0.0609, 0.1294	0.0491, 0.1026	0.0681, 0.1351

**Table 3:** Selected bond distances (Å) and bond angles (°) of zinc complexes **B**–**D**.

**Complex B**			
**Atoms**	**Distances (Å)**	**Atoms**	**Angles (°)**
Zn(1)-N(5)	2.158(4)	N(5)-Zn(1)-N(3)	94.01(15)
Zn(1)-N(3)	2.164(4)	N(5)-Zn(1)-N(2)	168.55(14)
Zn(1)-N(2)	2.190(3)	N(3)-Zn(1)-N(2)	94.32(14)
Zn(1)-N(1)	2.191(4)	N(5)-Zn(1)-N(1)	95.22(15)
Zn(1)-N(6)	2.201(4)	N(3)-Zn(1)-N(1)	92.50(14)
Zn(1)-N(4)	2.203(4)	N(2)-Zn(1)-N(1)	76.60(14)
N(2)-C(34)	1.273(6)	N(5)-Zn(1)-N(6)	77.26(15)
N(2)-C(35)	1.475(6)	N(3)-Zn(1)-N(6)	170.86(15)
N(4)-C(20)	1.271(7)	N(2)-Zn(1)-N(6)	94.70(14)
N(4)-C(21)	1.469(6)	N(1)-Zn(1)-N(6)	91.01(15)
N(6)-C(9)	1.265(6)	N(5)-Zn(1)-N(4)	94.20(14)
		N(3)-Zn(1)-N(4)	77.07(14)
		N(2)-Zn(1)-N(4)	95.30(14)
		N(1)-Zn(1)-N(4)	166.41(14)
		N(6)-Zn(1)-N(4)	100.62(14)
**Complex C**			
**Atoms**	**Distances (Å)**	**Atoms**	**Angles (°)**
Zn(1)-N(2)	2.172(3)	N(2)-Zn(1)-N(2)	98.5(1)
Zn(1)-N(1)	2.182(3)	N(2)-Zn(1)-N(1)	93.9(1)
N(2)-C(7)	1.482(4)	N(1)-Zn(1)-N(2)	93.9(1)
N(2)-C(6)	1.266(4)	N(1)Zn(1)-N(1)	91.8(1)
N(1)-C(5)	1.335(4)	N(1)-Zn(1)-N(2)	167.2(1)
N(3)-C(16)	1.142(7)	N(2)-Zn(1)-N(1)	76.6(1)
**Complex D**			
**Atoms**	**Distances (Å)**	**Atoms**	**Angles (°)**
Zn(1)-N(2)	2.181(4)	N(2)-Zn(1)-N(1)	92.4(1)
Zn(1)-N(1)	2.194(4)	N(1)-Zn(1)-N(2)	92.4(1)
N(2)-C(7)	1.480(6)	N(1)-Zn(1)-N(1)	90.0(1)
N(2)-C(6)	1.272(6)	N(1)-Zn(1)-N(2)	166.7(1)
C(17)-N(3)	1.09(2)	N(2)-Zn(1)-N(1)	77.0(1)
		N(2)-Zn(1)-N(2)	100.9(1)

**Table 4:** Non-covalent interactions in complexes **B**–**D**.

Complex	Centroid to Centroid (Å)	Vertical Distance (Å)	Displacement (Å)
**Complex B** Pyridine[N(1), C(29)-(33)]∙∙∙Phenyl[C(4), C(1), C(7), C(6), C(14)]	4.001(5)	3.660(6)	1.617(5)
Pyridine [N(3), C(15)-C(19)]∙∙∙Phenyl[C(37)-C(42)]	4.004(4)	3.303(7)	1.933(10)
Pyridine[N(5), C(1V), C(1K), C(2), C(8), C(12)]∙∙∙Phenyl[C(23)-C(28)]	3.774(4)	3.329(6)	1.628(11)
**Complex C** Pyridine[N(1), C(1)-C(5)∙∙∙Phenyl[C(9)-C(14)]	4.02156(5)	3.47235(5)	2.02873(3)
**Complex D** Pyridine[N(1), C(1)-C(5)∙∙∙Phenyl[C(9)-C(14)]	4.173(4)	3.493(7)	2.283(10)

**Table 5:** Minimal inhibitory concentrations (mg/mL) of coordination compounds **A**–**D**.

	*E. coli*	*P. aeruginosa*	*S. aureus*
**A**	3.12	3.12	1.56
**B**	12.5	12.5	3.12
**C**	6.25	6.25	1.56
**D**	6.25	6.25	6.25
**Penicillin**	3.12	1.56	0.78

**Table 6:** Median lethal concentration (LC_50_) and median lethal dosage (LD_50_) of complexes **A**–**D** against *A. salina* lethality assay and intraperitoneal toxicity evaluation.

Assay	Complex A	Complex B	Complex C	Complex D
Brine Shrimp (LC_50_)	414 ± 1.32 µM	564.5 ± 0.25 µM	385 ± 0.60 µM	350 ± 2.8 µM
Acute IP Toxicity study (LD_50_)	209.17 mg/kg	295.80 mg/kg	500 mg/kg	223.61 mg/kg

**Table 7:** Mortality of mice obtained after 48 h of i.p. administration of complexes **A**–**D** at different doses.

Complex	Dose (mg/kg)	Number of Dead Animals After 48 h of Administration
**A**	10	0
	100	0
	175	1
	250	3
	500	3
**B**	10	0
	100	0
	175	2
	250	2
	500	3
**C**	10	0
	100	1
	500	1
**D**	10	0
	100	1
	500	3

## Data Availability

Data are contained within the article and Supplementary Materials.

## Supplementary Materials

The following supporting information can be downloaded at: https://www.mdpi.com/article/10.3390/molecules29235555/s1. Crystallographic data for complexes **B**, **C**, and **D** have been deposited with the Cambridge Crystallographic Data Centre as supplementary publication nos. CCDC 2065362, 2381803, and 2381809. Copies of the data can be obtained free of charge from the Director, CCDC, 12 Union Road, Cambridge CB2 1EZ, UK (fax: +44-1223-336-033; e-mail: deposit@ccdc.cam.ac.uk; http://www.ccdc.cam.ac.uk (Accessed on 3 September 2024).
