# Supplementary material for: Synthesis, Crystal Structures, Antimicrobial Activity, and Acute Toxicity Evaluation of Chiral Zn(II) Schiff Base Complexes
Source: Molecules. 2024 Nov 25;29(23):5555. doi: 10.3390/molecules29235555 (PMC11643661; doi:10.3390/molecules29235555)
Supplement: Supplementary file 1 [file molecules-29-05555-s001.zip › supplementary Molecules Zn R1.pdf]

Synthesis, crystal structures, antimicrobial activity, and acute toxicity evaluation of chiral Zn(II) Schiff base complexes

*Daniela Gutiérrez Arguelles [1], Claudia P. Villamizar [2], Eduardo Brambila-Colombres[1], Bertin Anzaldo [1], Angel Mendoza [3], Guadalupe Hernández Téllez [1,] * and Pankaj Sharma [2,]**

[1]Lab. Síntesis de Complejos. Fac. Ciencias Químicas, Universidad Autónoma de Puebla, Edif. FCQ-6, C.U. Av. San Claudio y Blvd. 14 Sur, Col. San Manuel, C.P. 72592, Puebla, Pue. México. [2]Instituto de Química-UNAM, Circuito exterior, C.U. Coyoacán, C.P. 04510, México, CDMX. [3]Centro de Química del Instituto de Ciencias, Benemérita Universidad Autónoma de Puebla, 18 Sur y Av. San Claudio, Col. San Manuel, Puebla 72570, México

Supplementary Information

1. Imine a

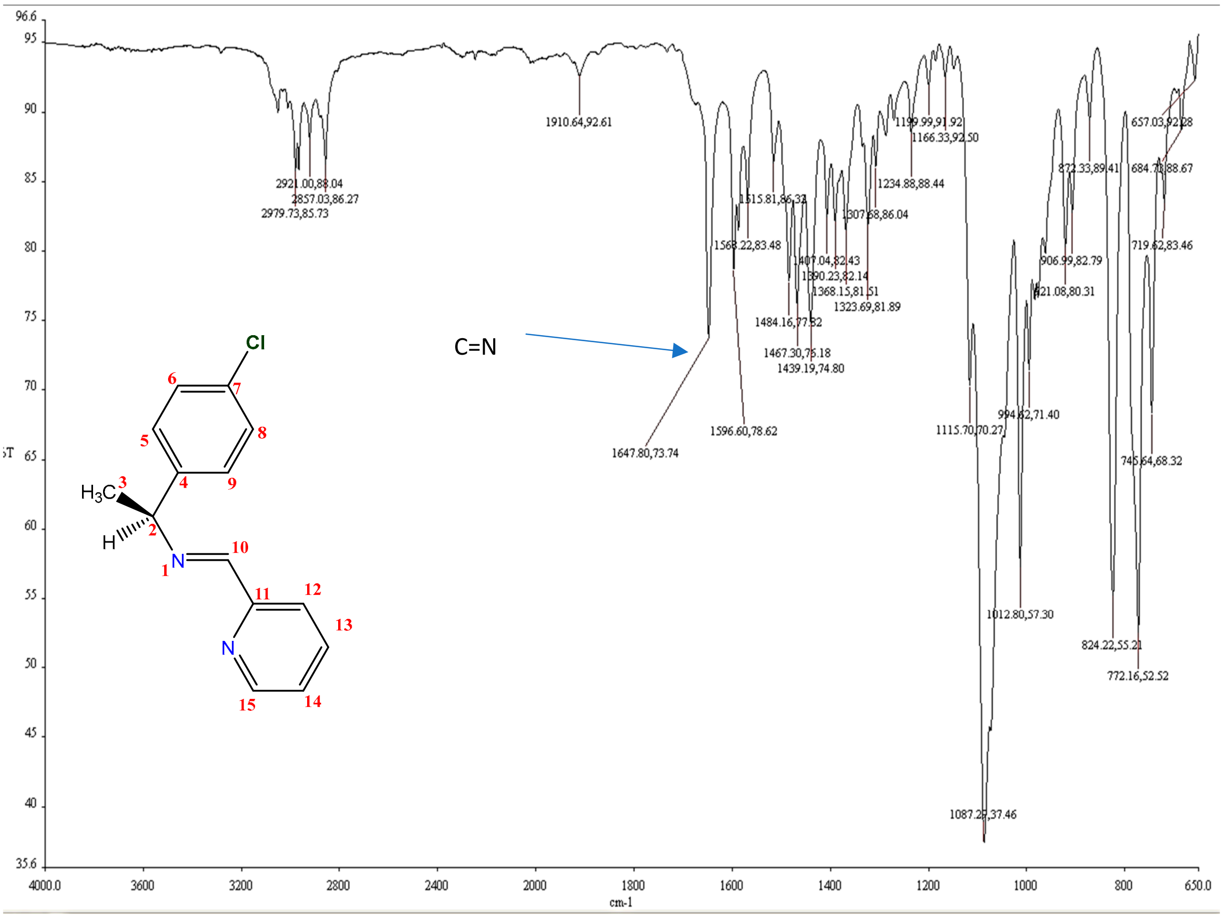

Figure S1. FT-IR spectrum of imine-a

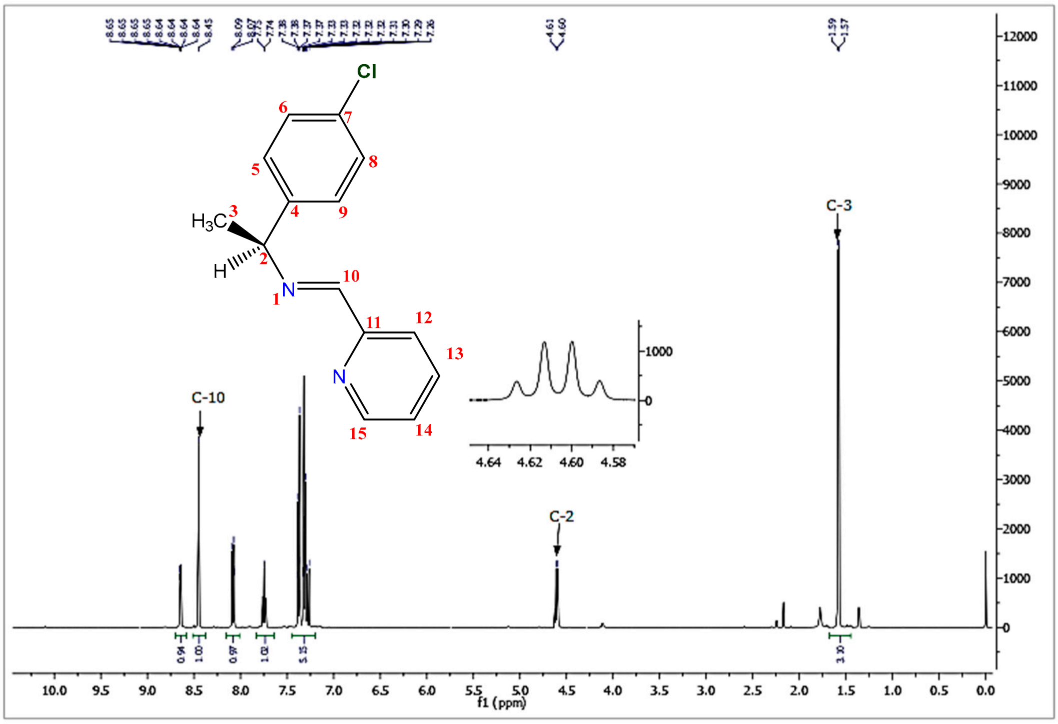

Figure S2. ^{1}H-NMR(400 MHz, $CDCl_3$) spectrum of imine-**a**

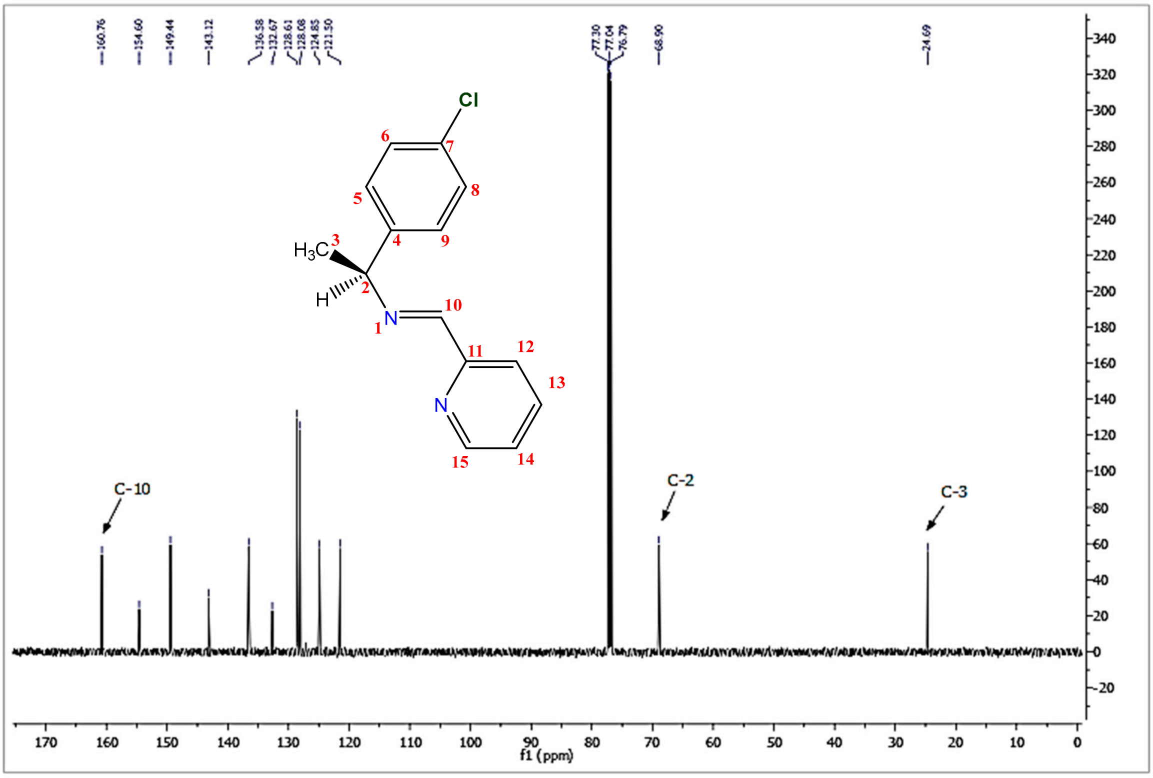

Figure S3. ^{13}C-NMR (100 MHz, $CDCl_3$) spectrum of imine-**a**

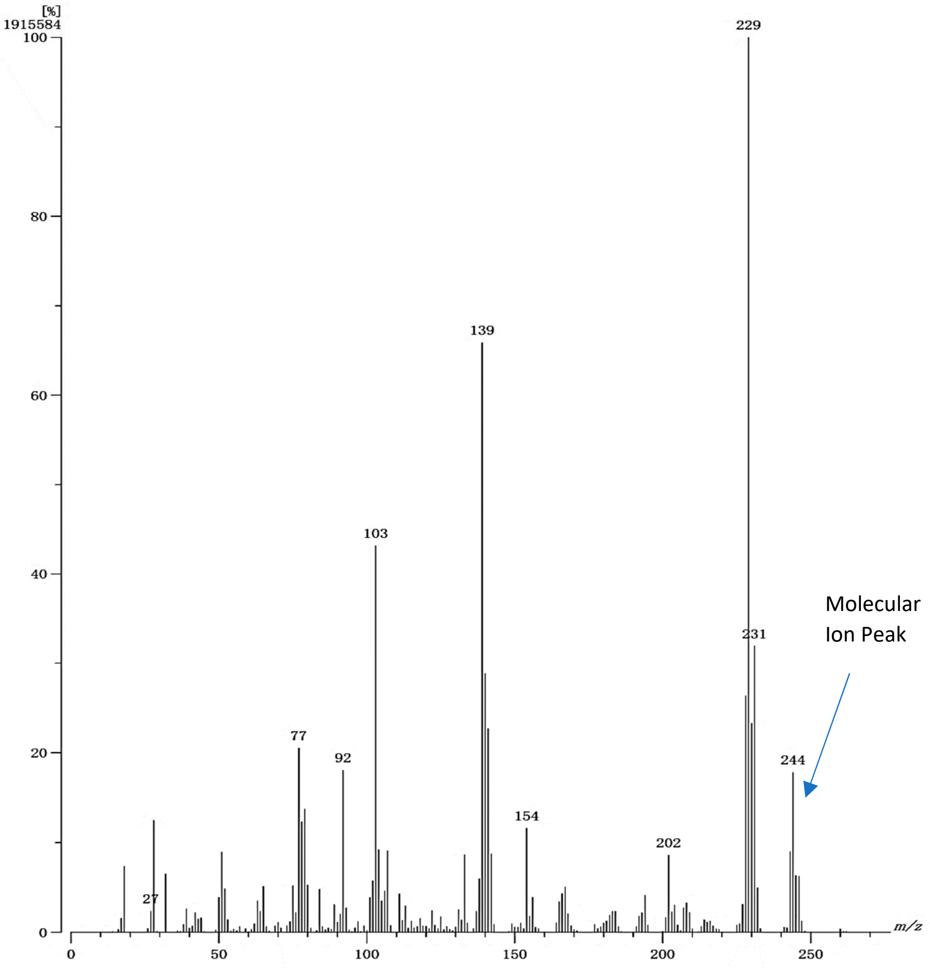

Figure S4. EI mass spectrum of imine-**a** in the positive-ion mode

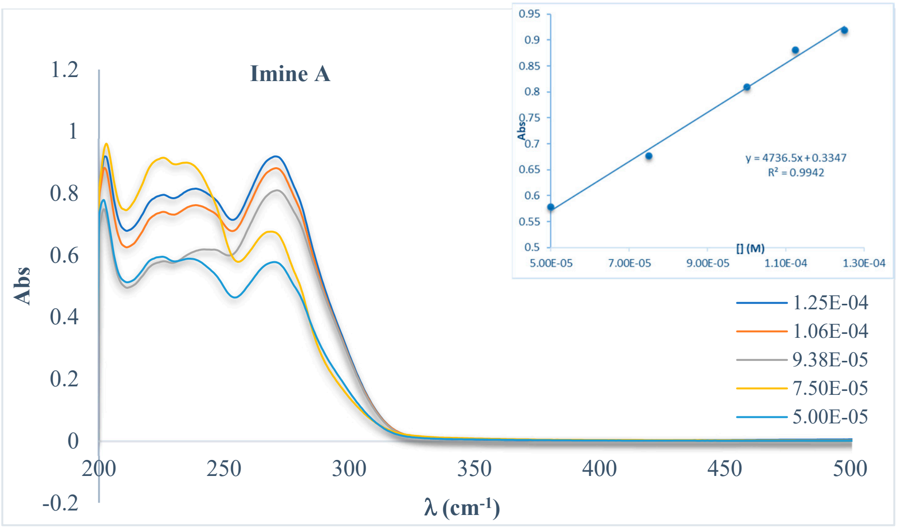

Figure S5. UV-Vis spectra (ACN) of imine **a**

1. Imine b

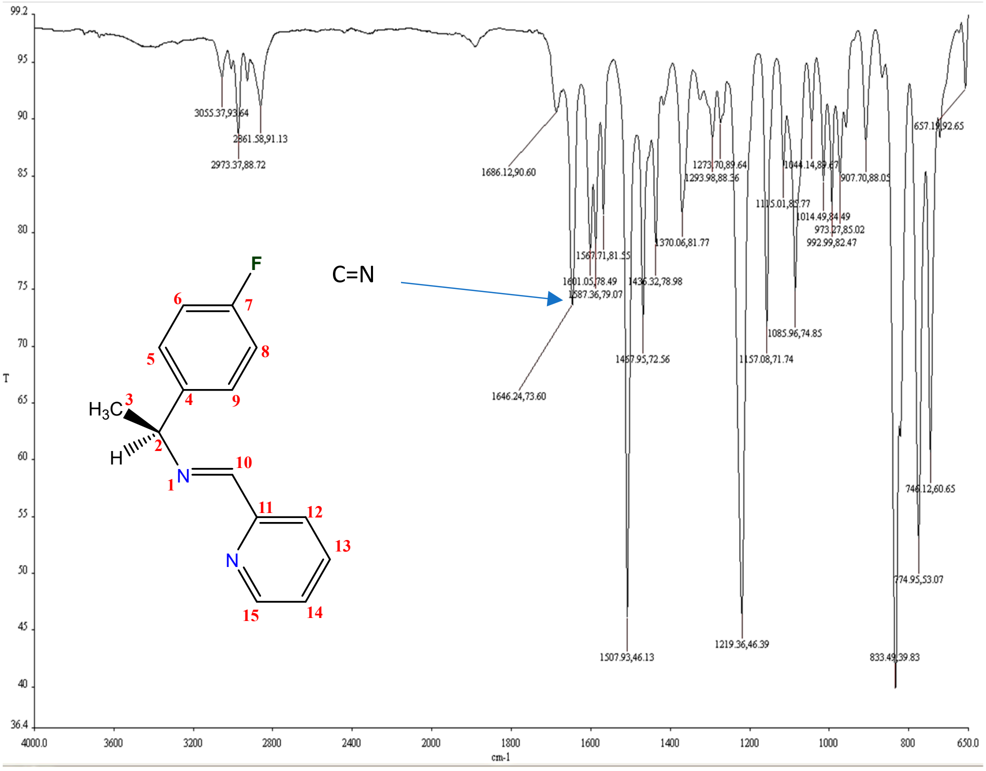

Figure S6. FT-IR spectrum of imine-**b**

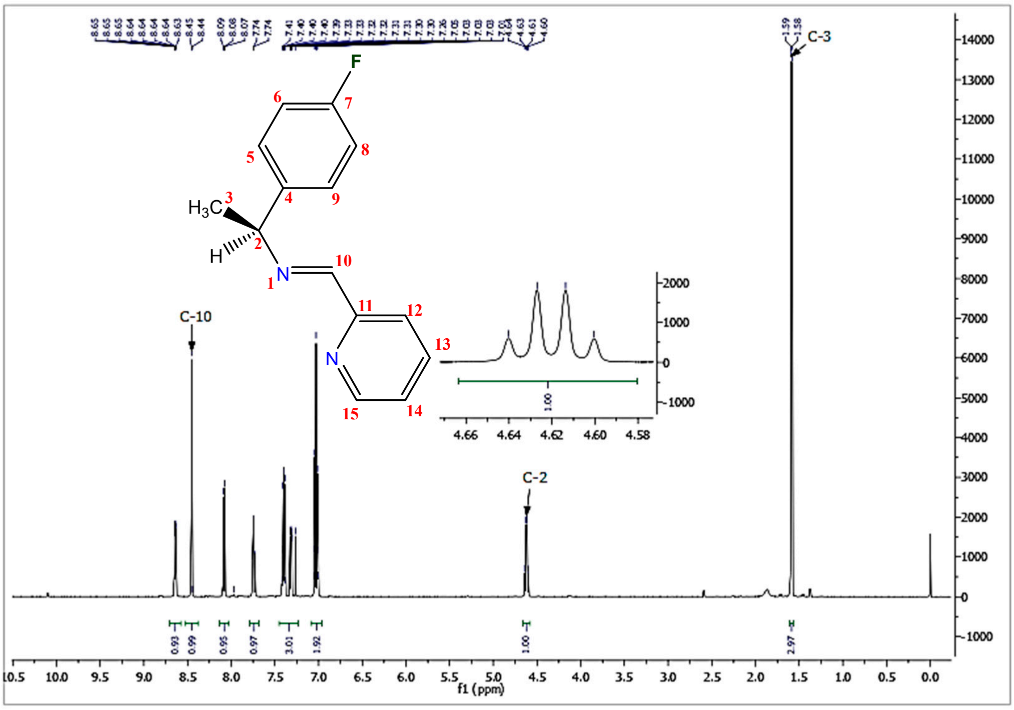

Figure S7. ^{1}H-NMR (400 MHz, $CDCl_3$) spectrum of imine-**b**

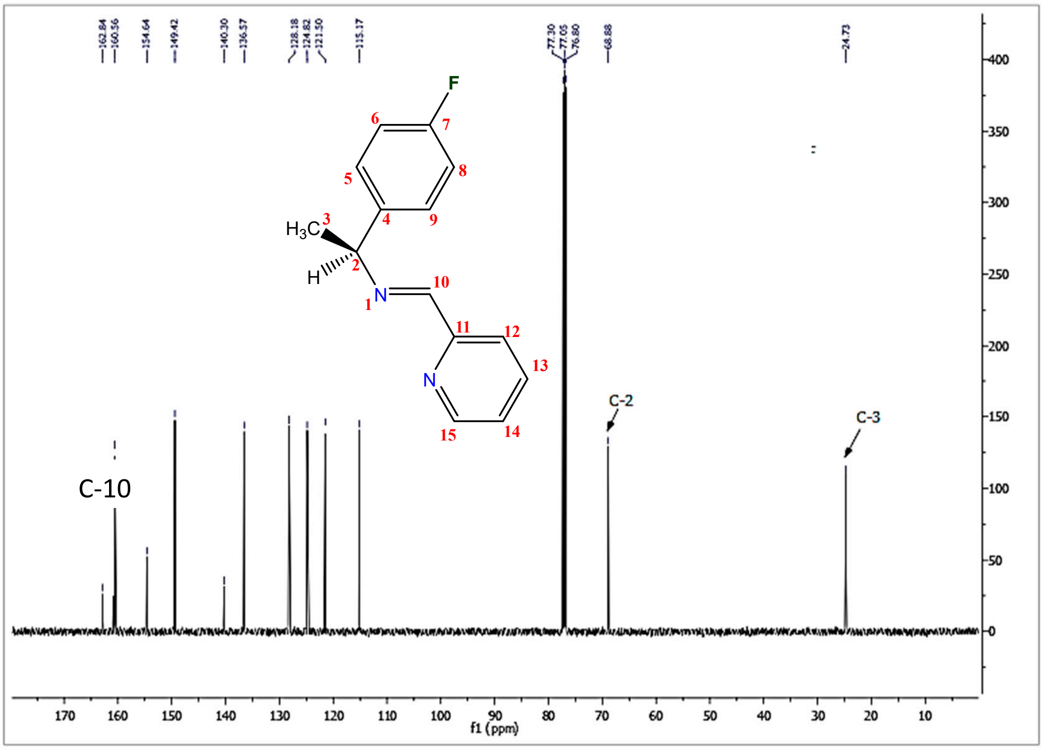

Figure S8. ^{13}C-NMR(100 MHz, $CDCl_3$) spectrum of imine-**b**

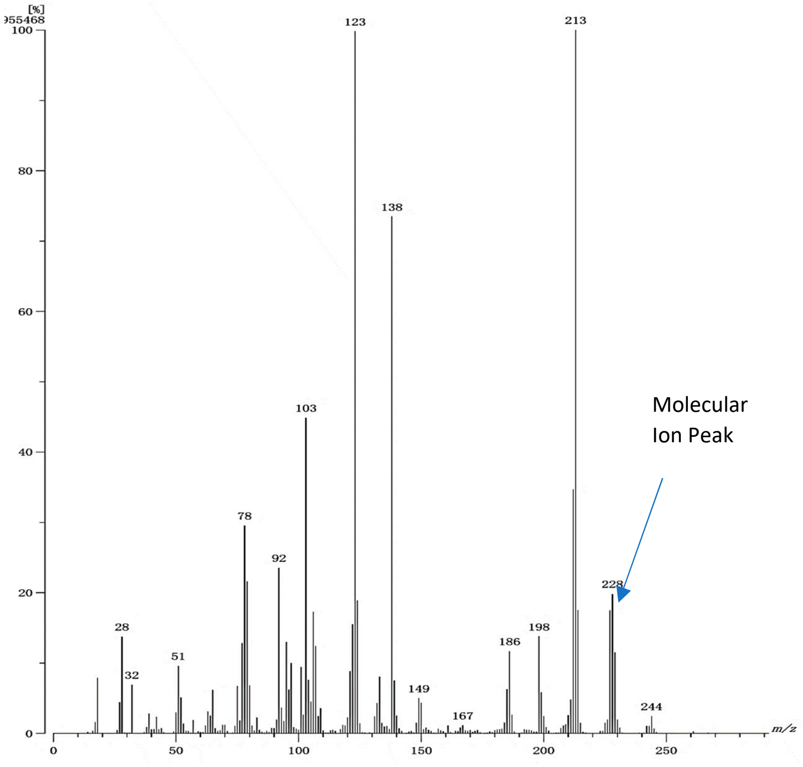

Figure S9 EI mass spectrum of imine-**b** in the positive-ion mode

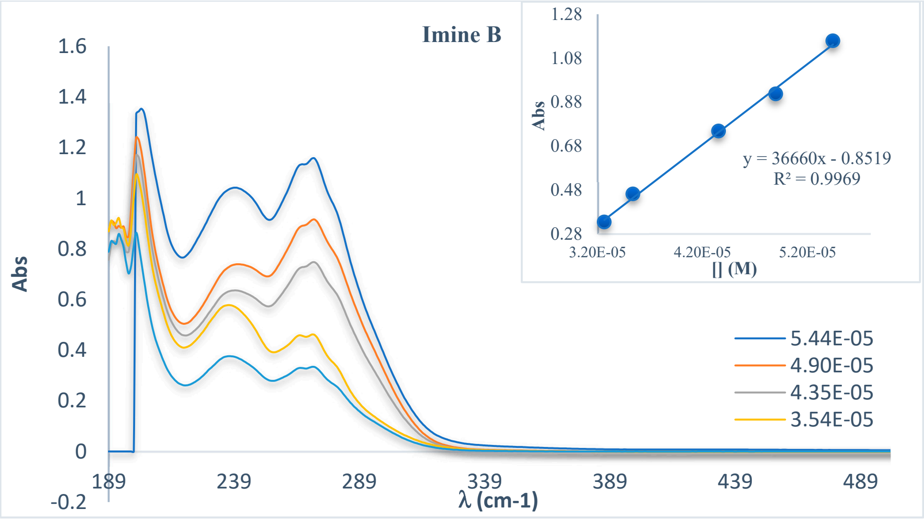

Figure S10. UV-Vis spectra (ACN) of imine **b**

2. Imine c

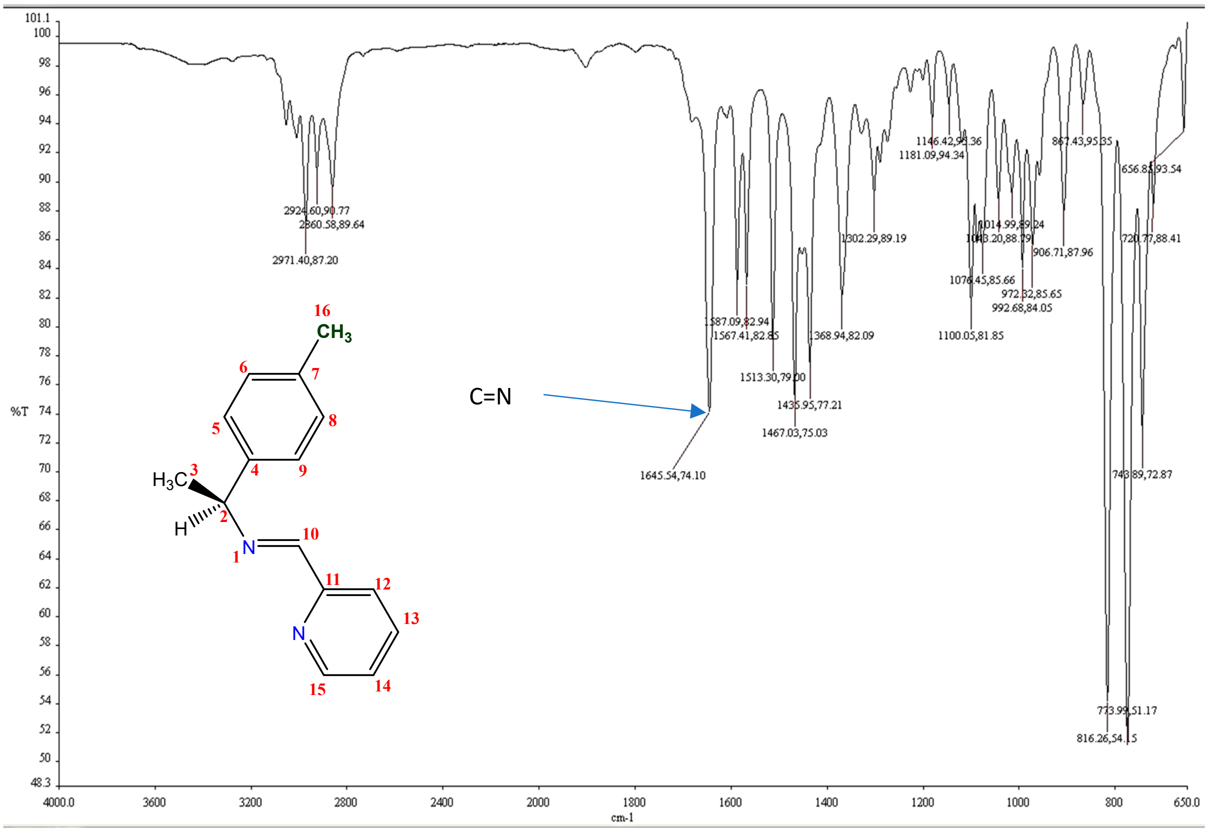

Figure S11. FT-IR spectrum of imine-**c**

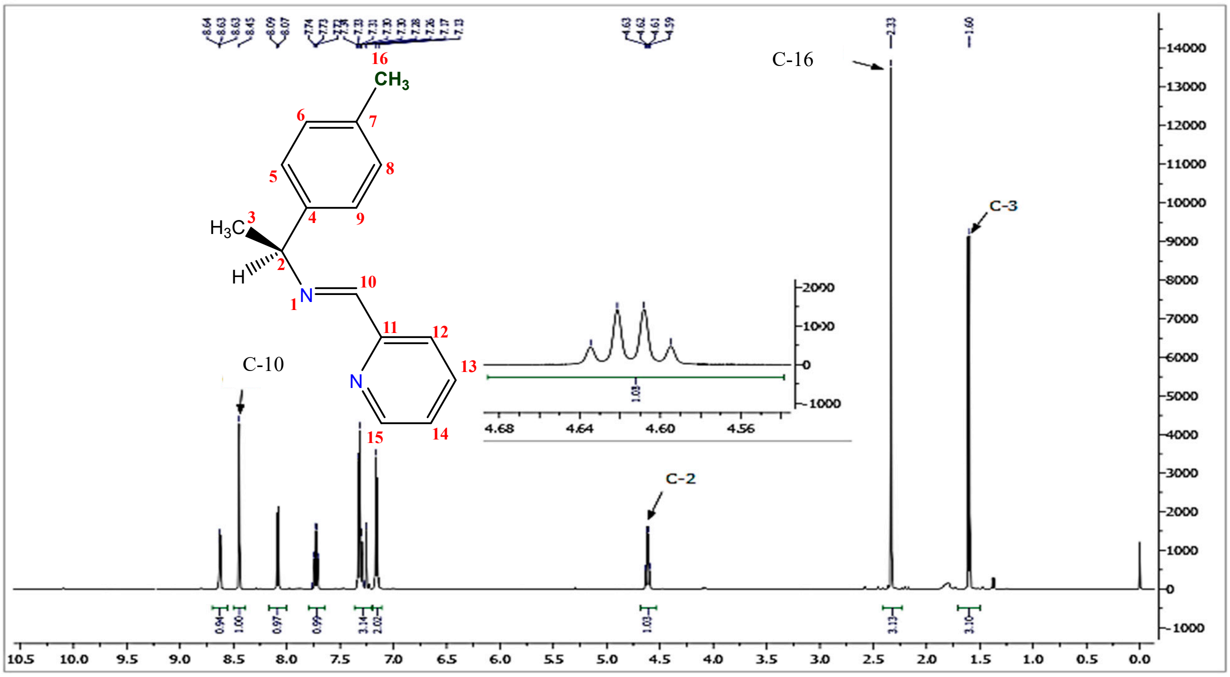

Figure S12. ¹H-NMR(400 MHz, $CDCl_3$) spectrum of imine-**c**

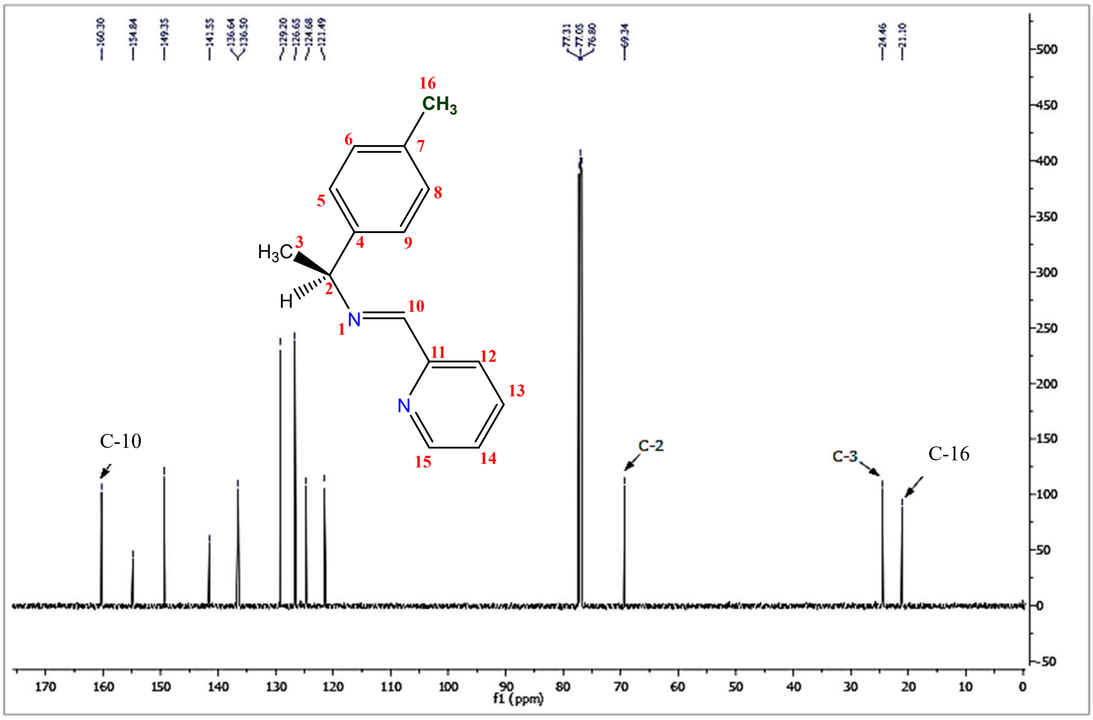

Figure S13. ¹³C-NMR (100 MHz, $CDCl_3$) spectrum of imine-**c**

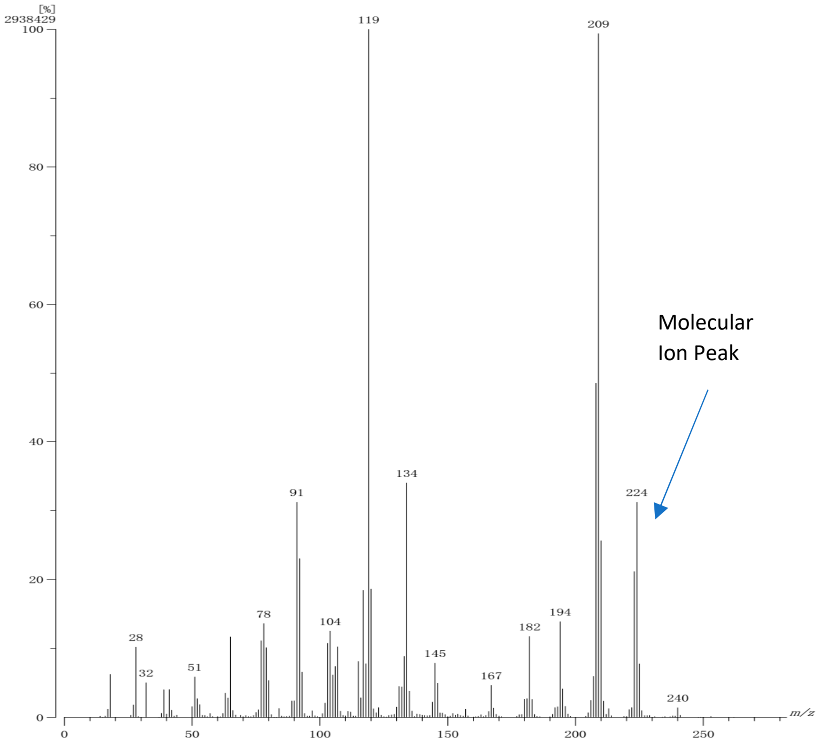

Figure S14. EI mass spectrum of imine-**c** in the positive-ion mode

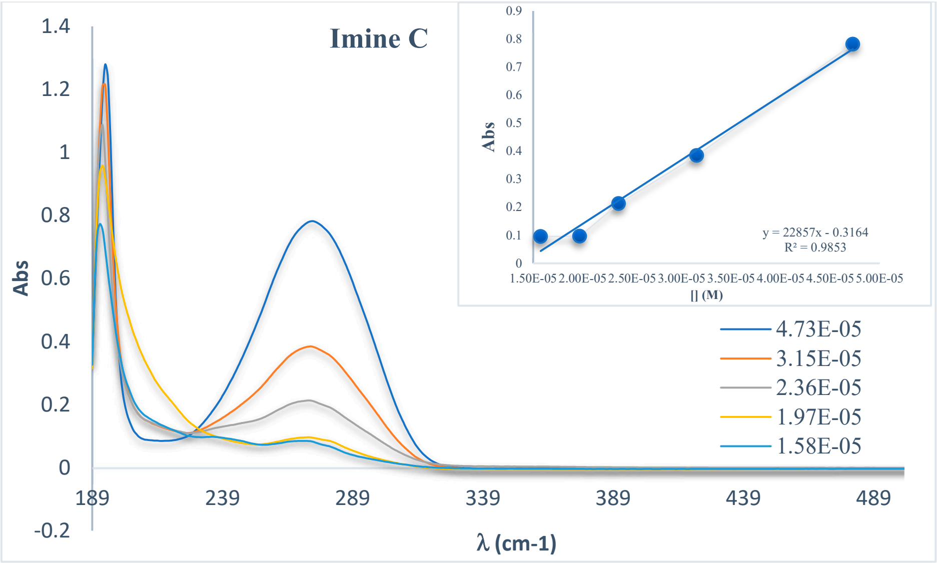

Figure S15. UV-Vis spectra (ACN) of imine **c**

3. Imine d

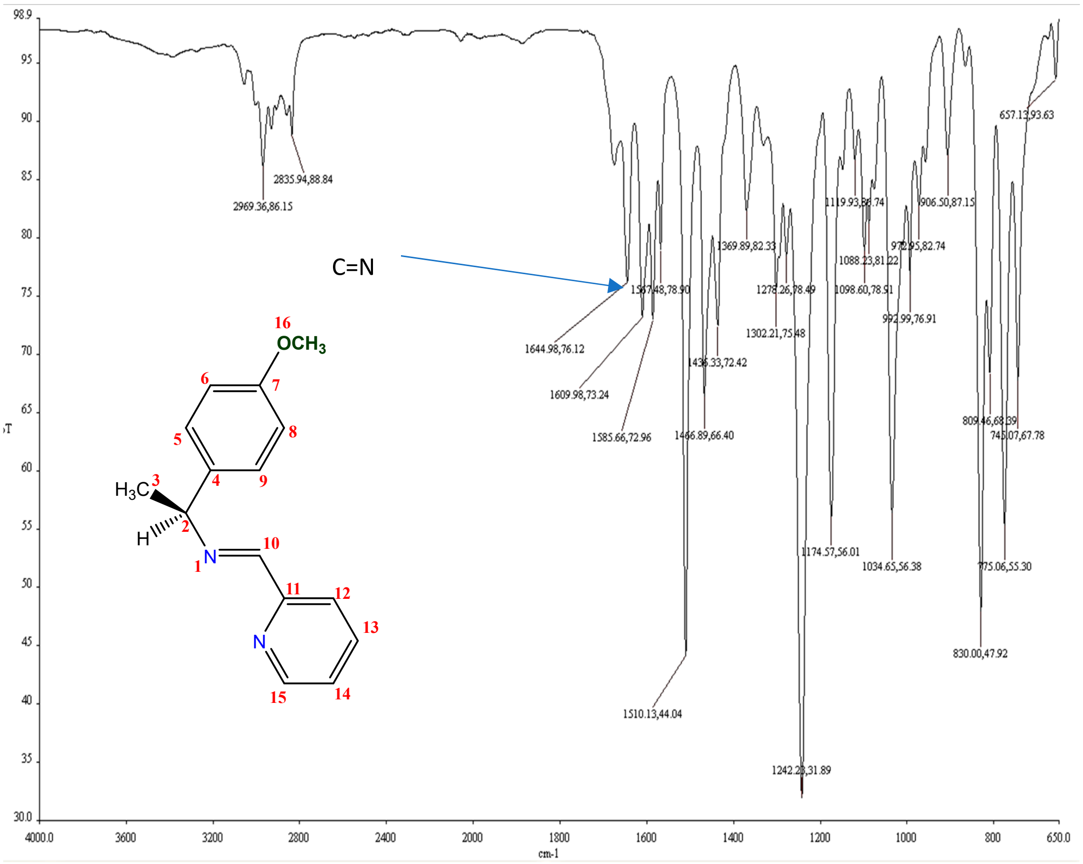

Figure S16. FT-IR spectrum of imine-**d**

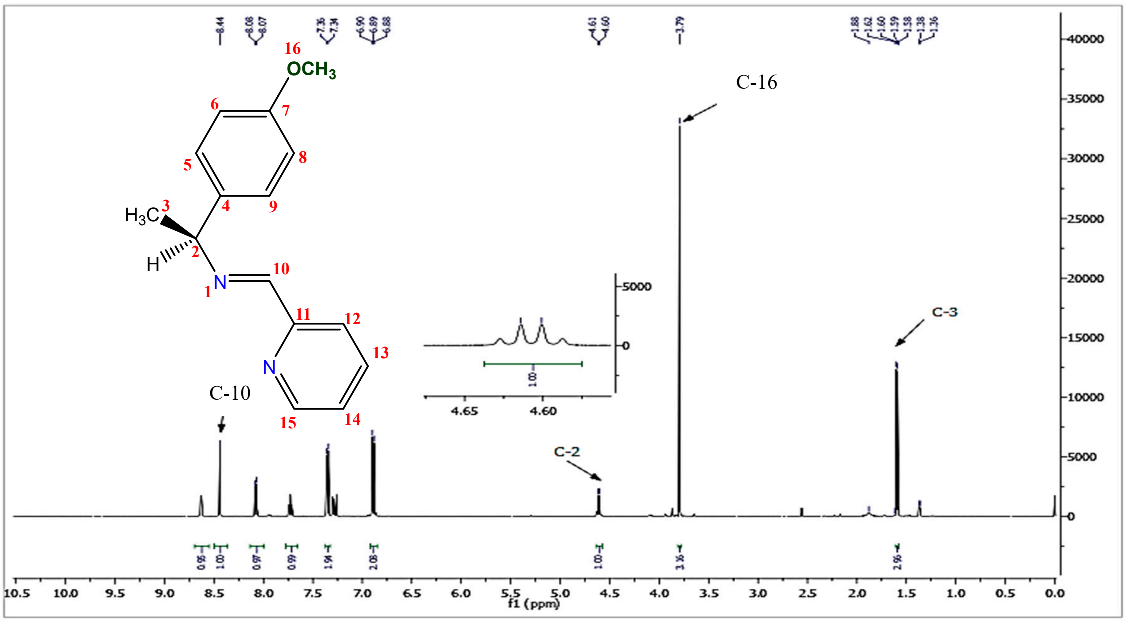

Figure S17. ^{1}H-NMR(400MHz, $CDCl_3$) spectrum of imine-**d**

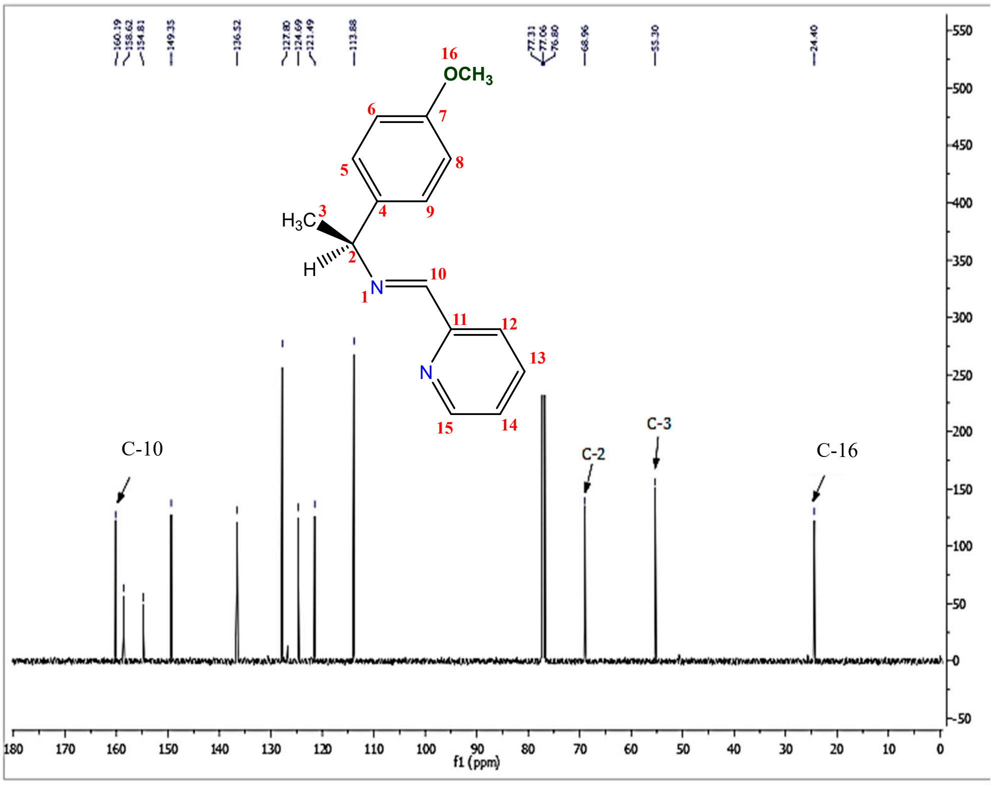

Figure S18. ^{13}C-NMR(100 MHz, $CDCl_3$) spectrum of imine-**d**

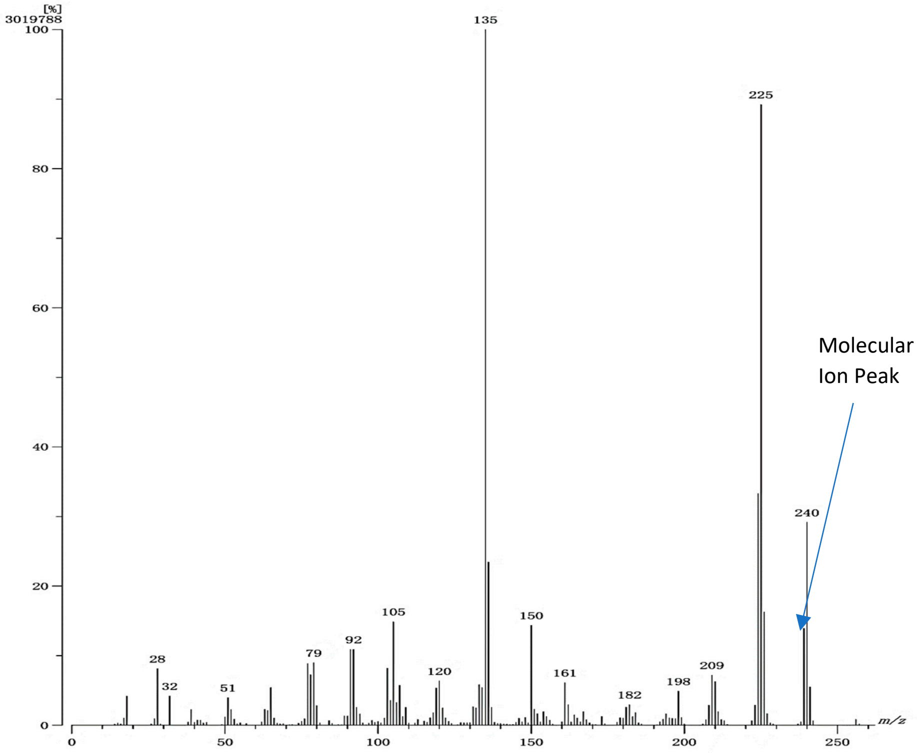

Figure S19. EI mass spectrum of imine-**d** in the positive-ion mode

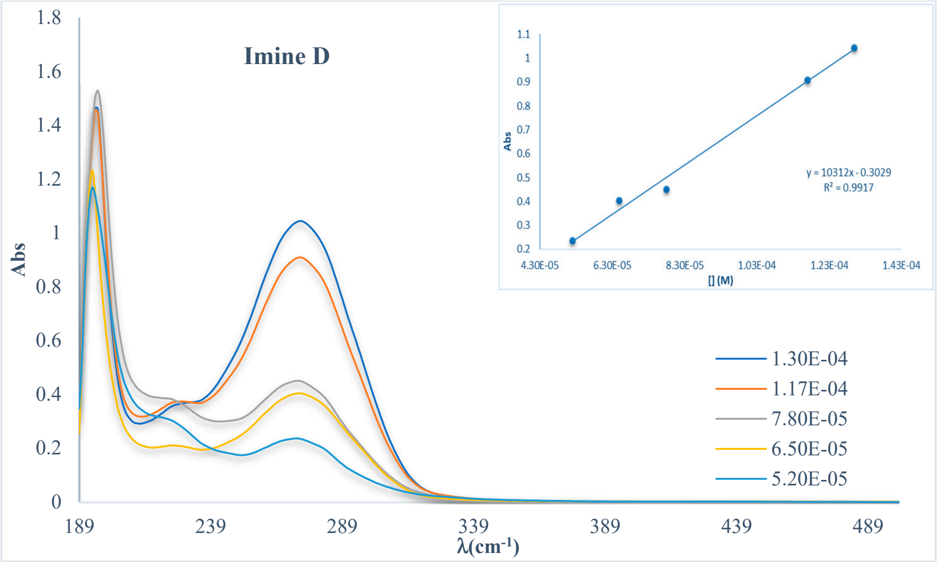

Figure S20. UV-Vis spectra (ACN) of imine **d**

4. Complex A

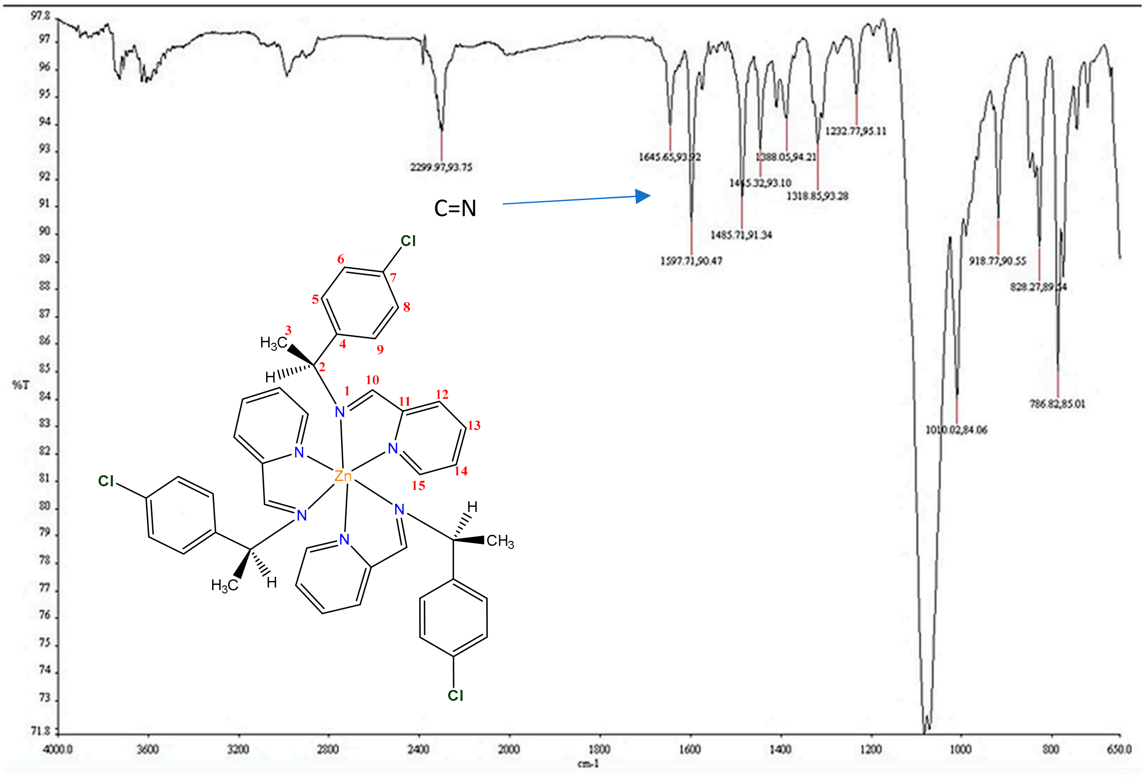

Figure S21. FT-IR spectrum of complex **A**

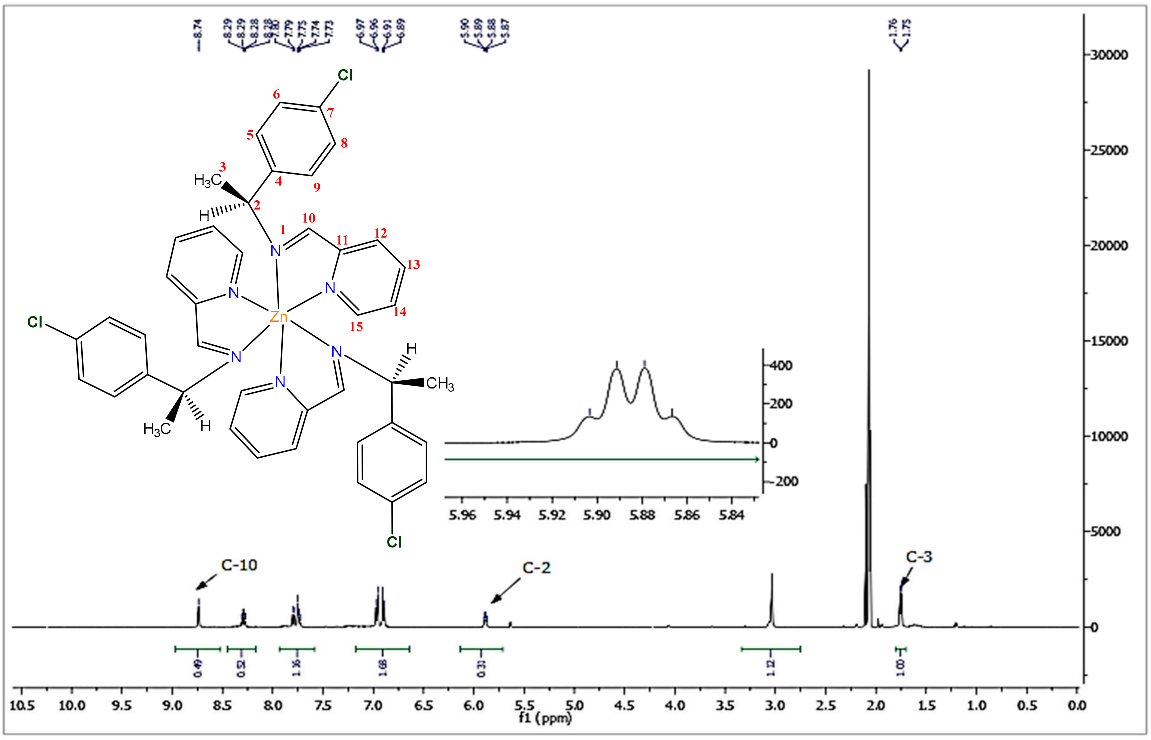

Figure S22. ^{1}H-NMR(400 MHz, C_3D_6O) spectrum of complex **A**

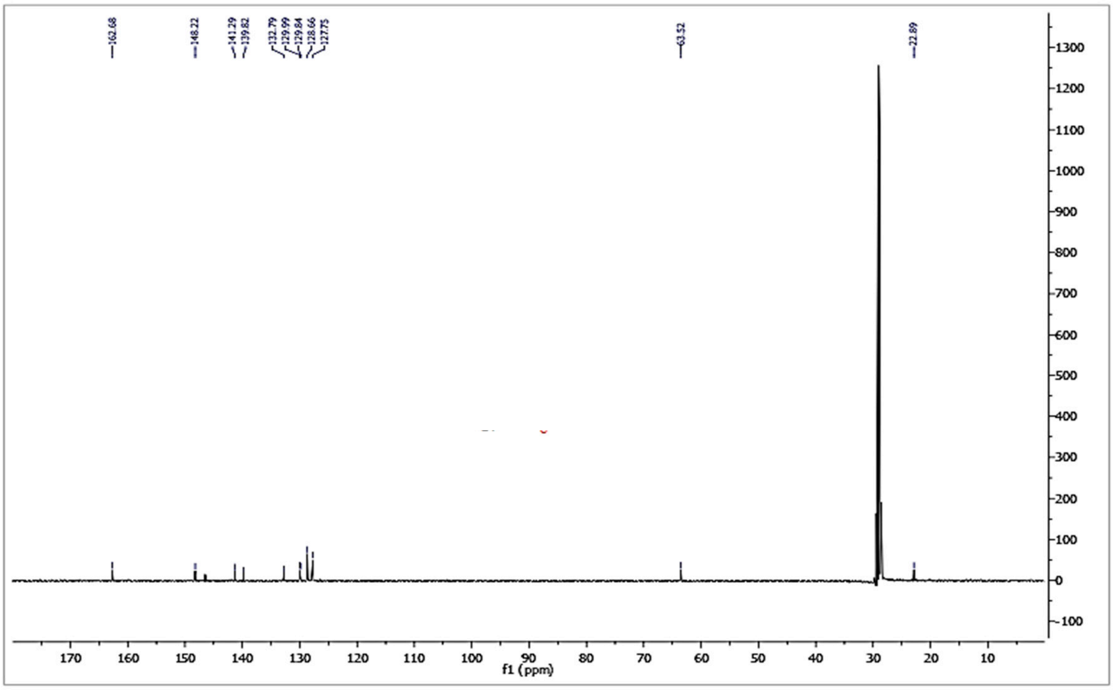

Figure S23. ^{13}C-NMR (100 MHz, C_3D_6O) spectrum of complex **A**

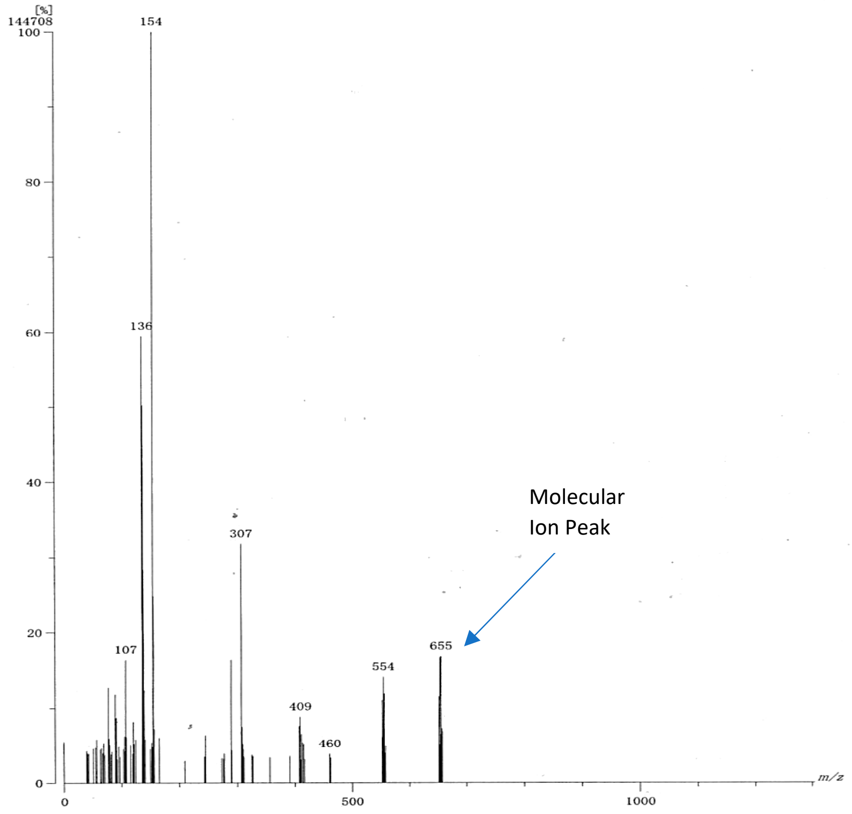

Figure S24. DART mass spectrum of complex **A** in the positive-ion mode

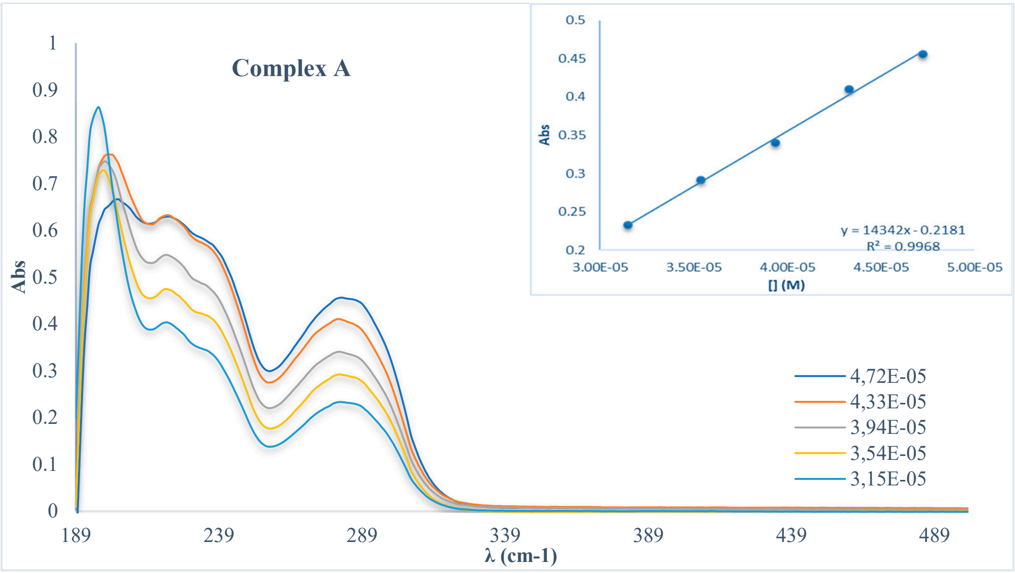

Figure S25. UV-Vis spectra (ACN) of complex **A**

6 Complex B

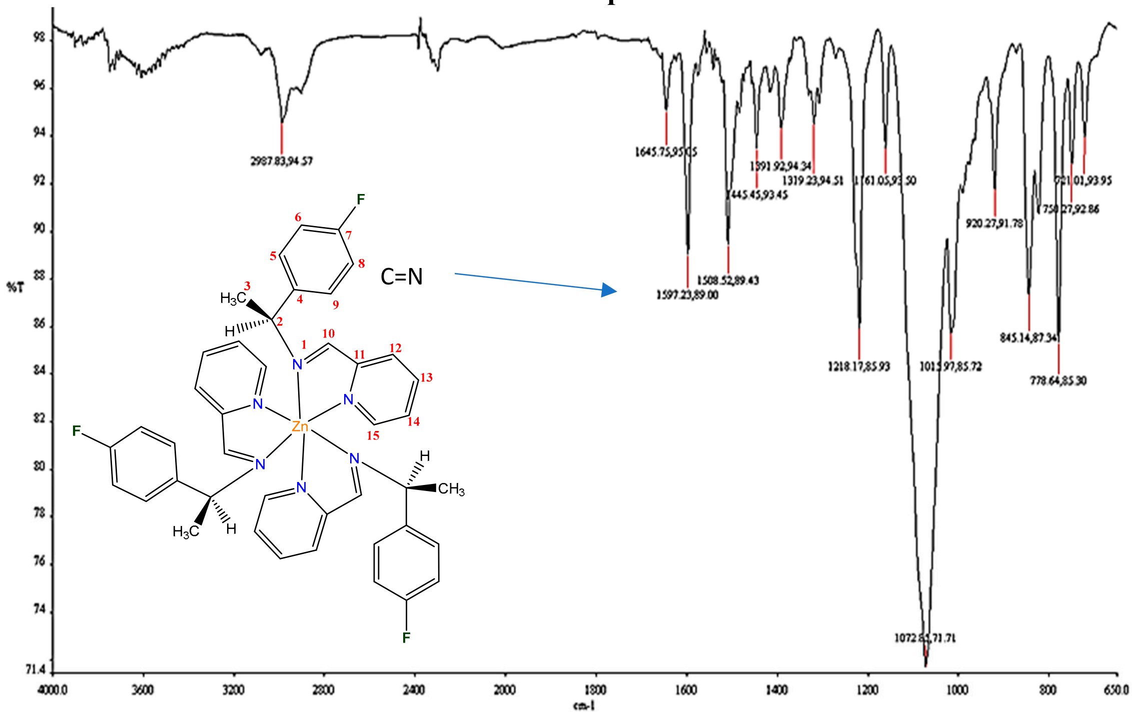

Figure S26. FT-IR spectrum of complex **B**

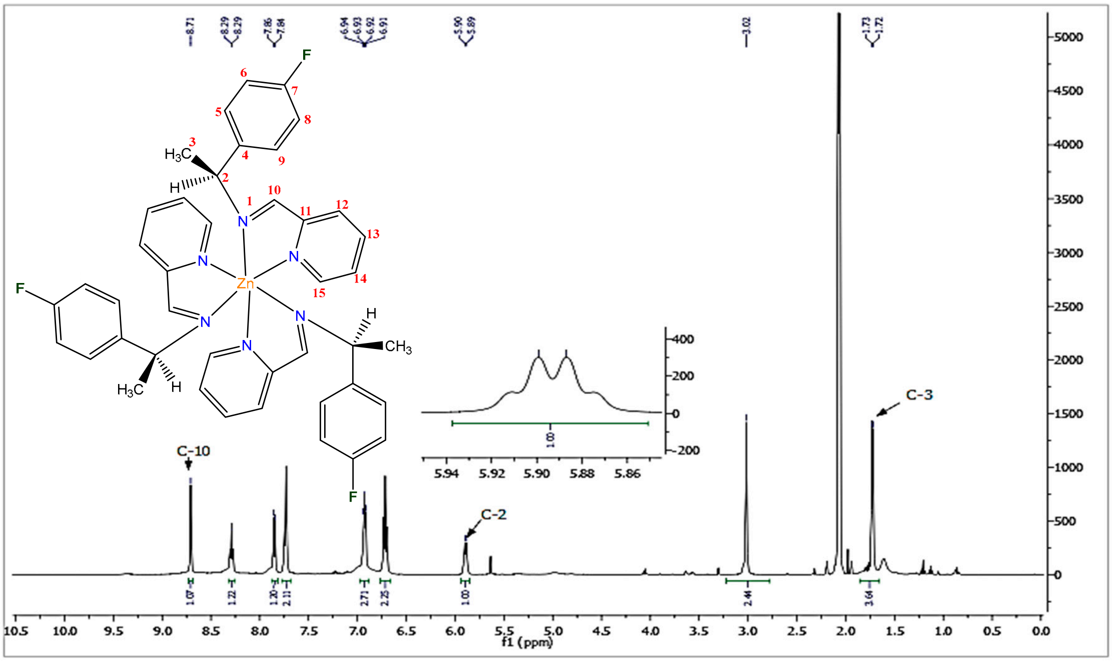

Figure S27. ^{1}H-NMR(400 MHz, C_3D_6O) spectrum of complex **B**

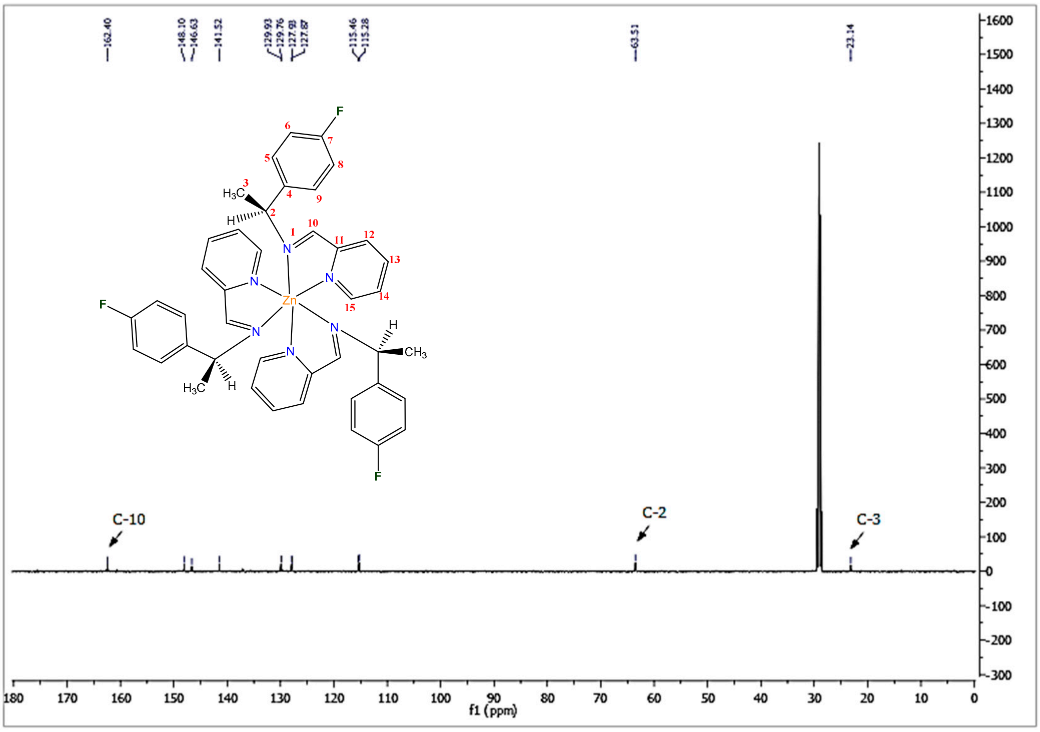

Figure S28. ^{13}C-NMR(100 MHz, C_3D_6O) spectrum of complex **B**

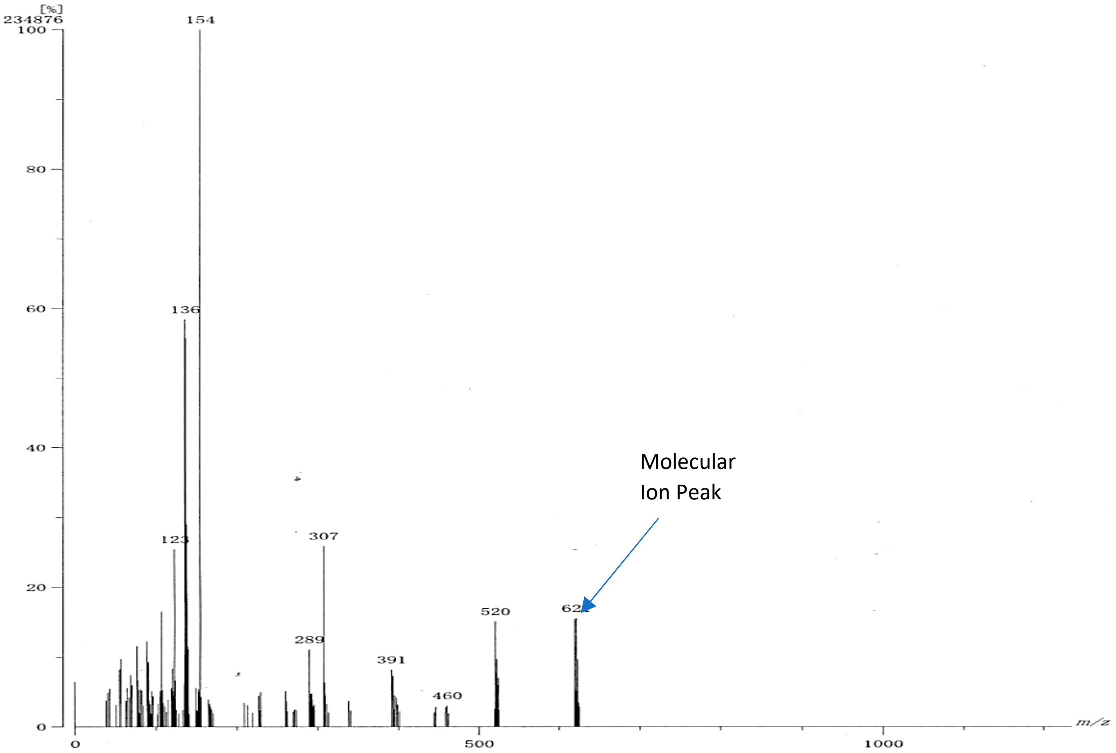

Figure S29. DART mass spectrum of complex **B** in the positive-ion mode.

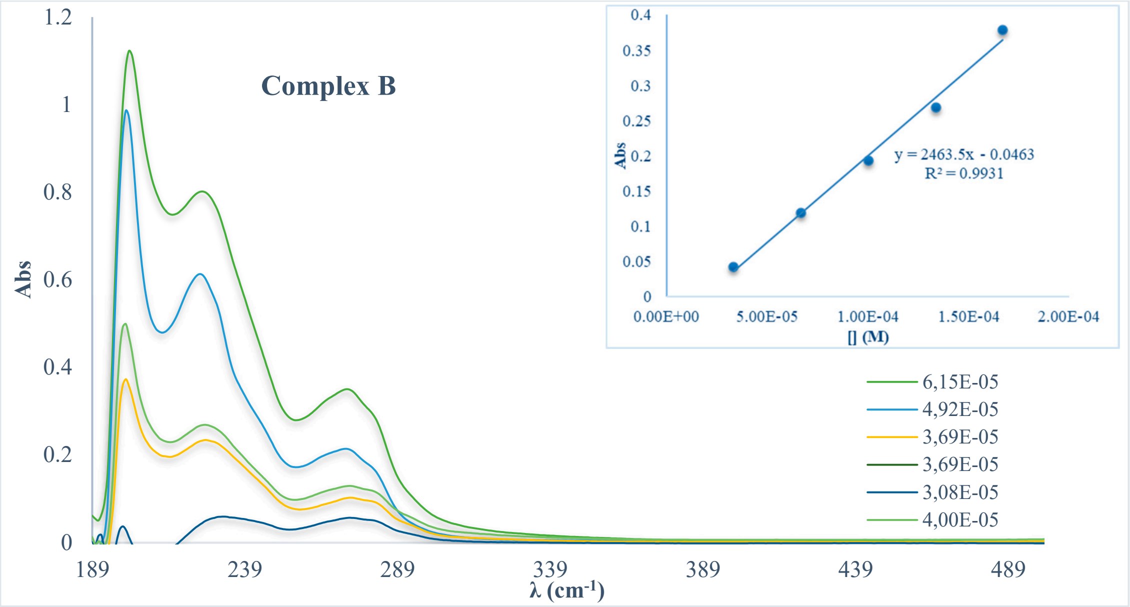

Figure S30. UV-Vis spectra (ACN) of complex **B**

7. Complex C

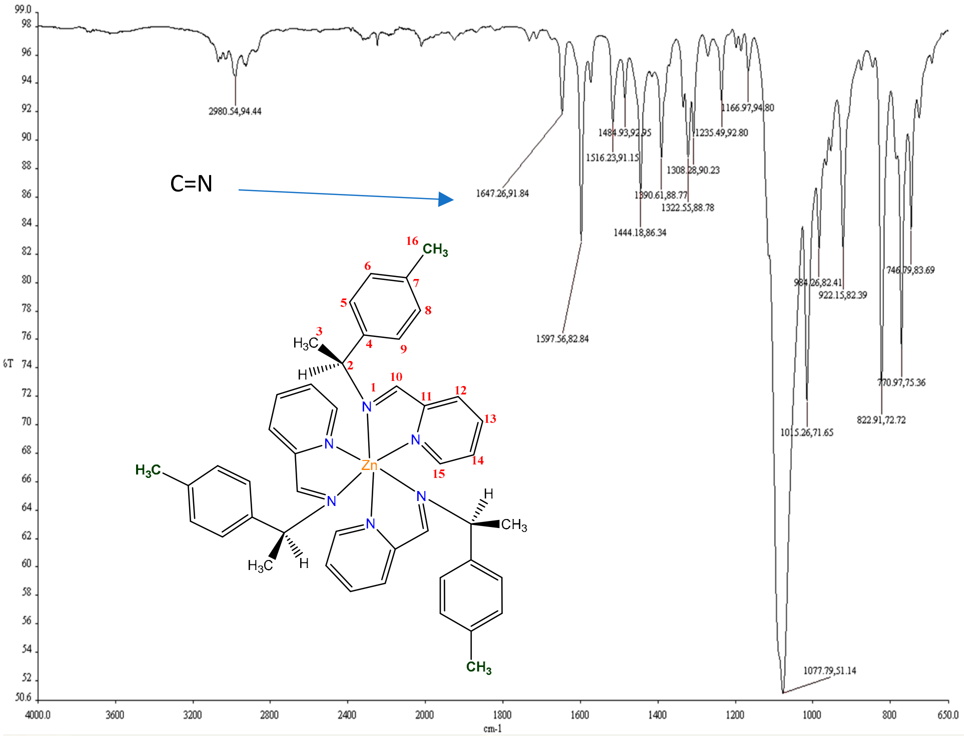

Figure S31. FT-IR spectrum of complex **C**

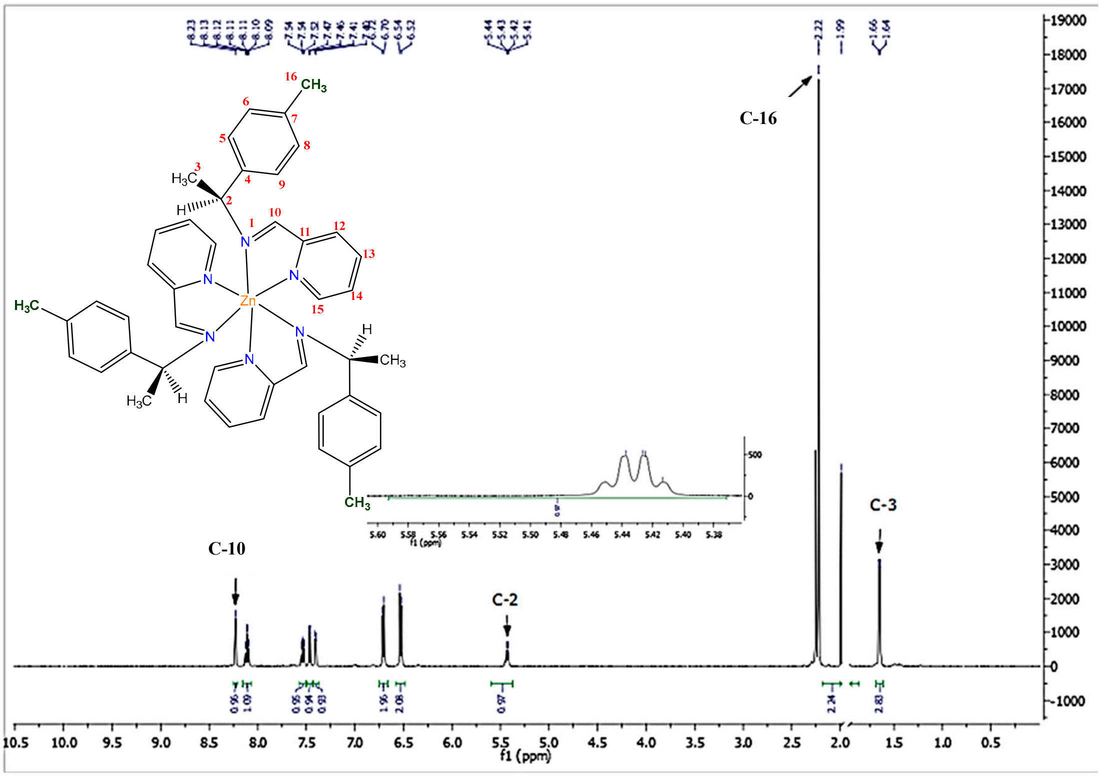

Figure S32. ^{1}H-NMR (400 MHz, C_3D_6O) spectrum of complex **C**

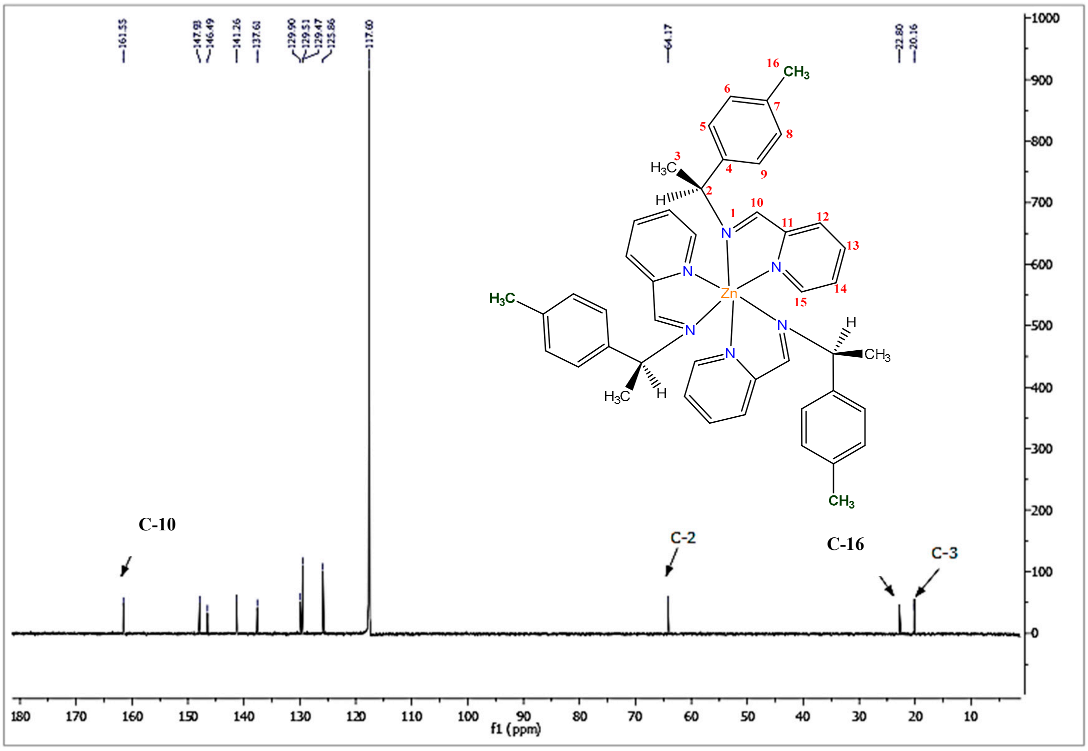

Figure S33. ^{13}C-NMR (100 MHz, CD_3CN) spectrum of complex **C**

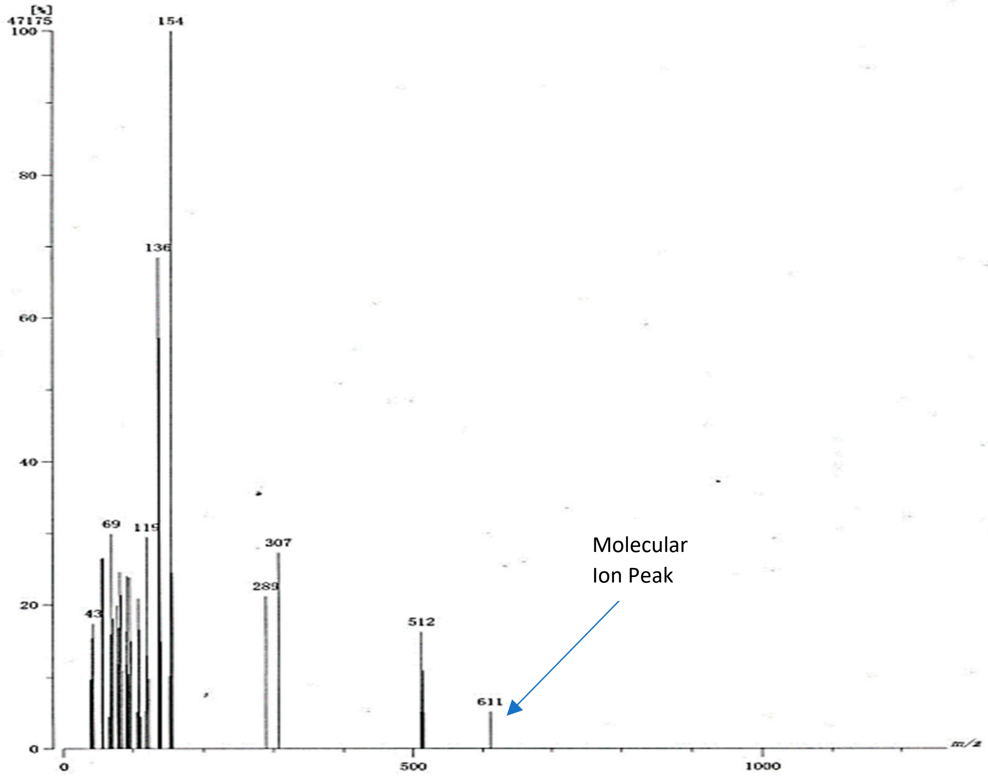

Figure S34. DART mass spectrum of complex **C** in the positive-ion mode

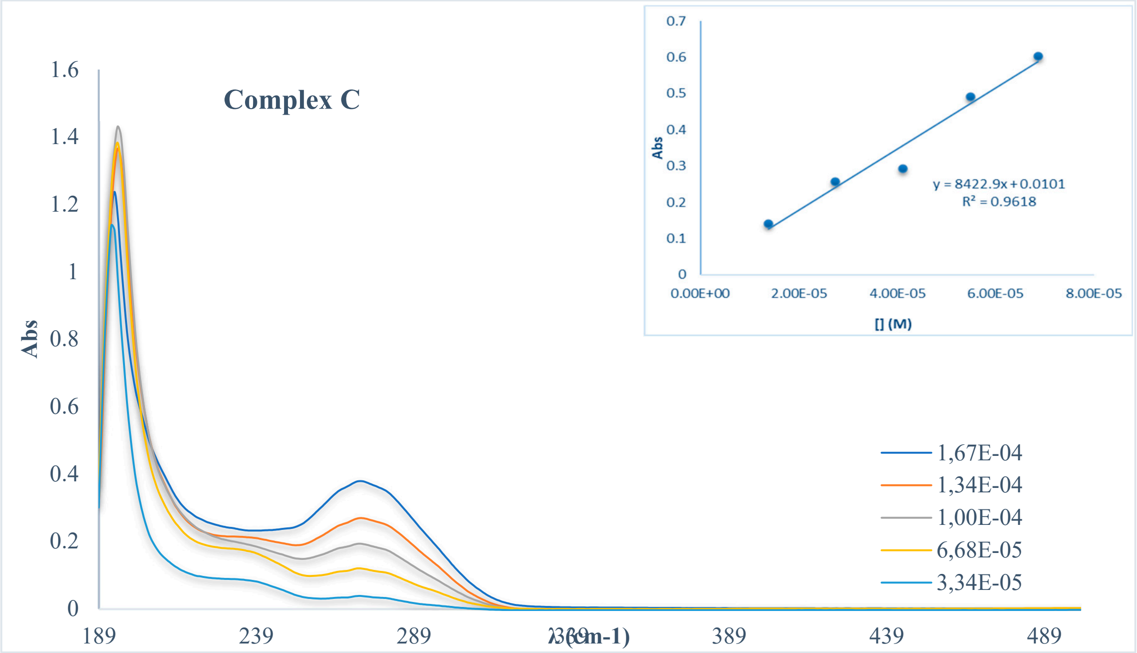

Figure S35. UV-Vis spectra (ACN) of complex **C**

8. Complex D

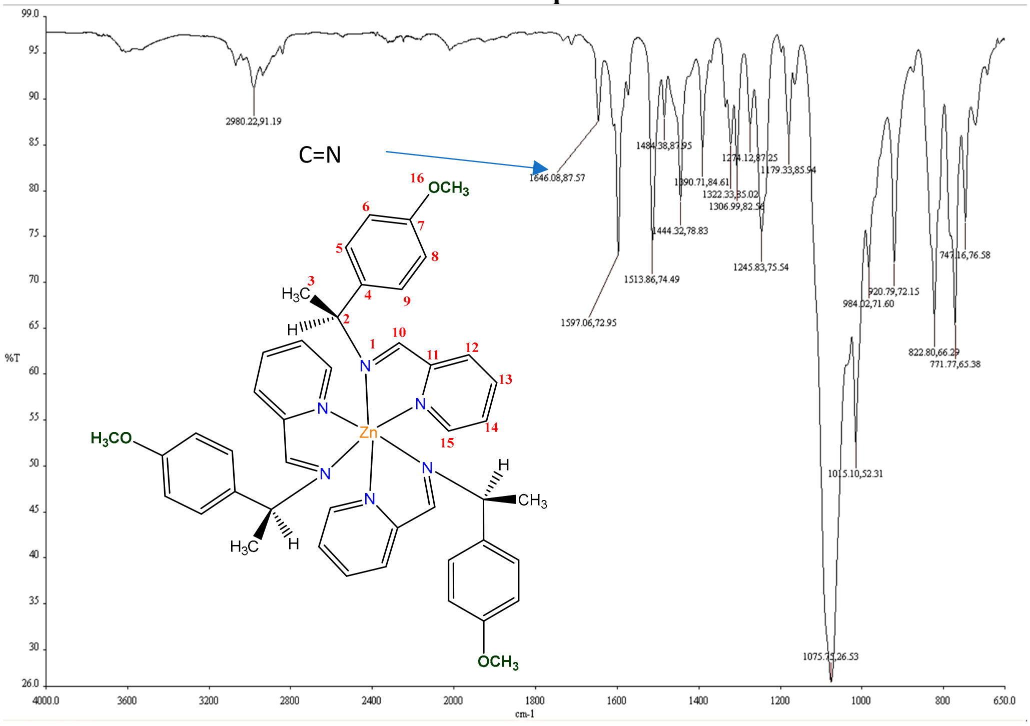

Figure S36. FT-IR spectrum of complex **D**

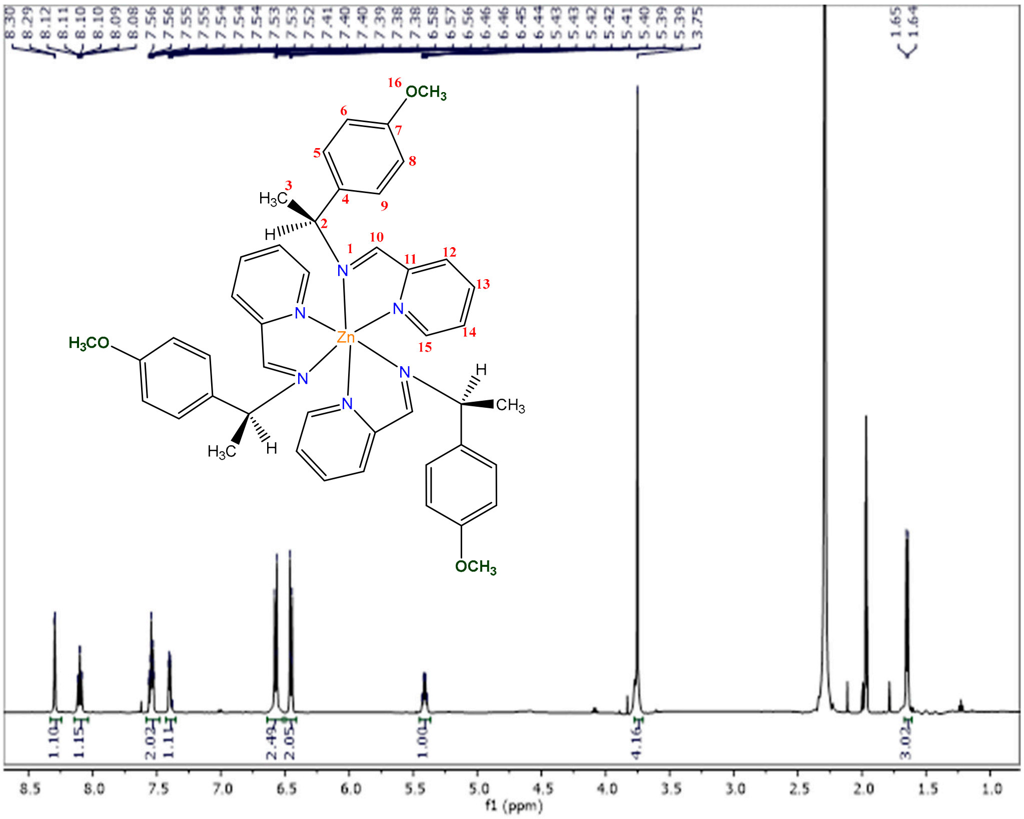

Figure S37 ^{1}H-NMR (400 MHz, C_3D_6O) spectrum of complex **D**

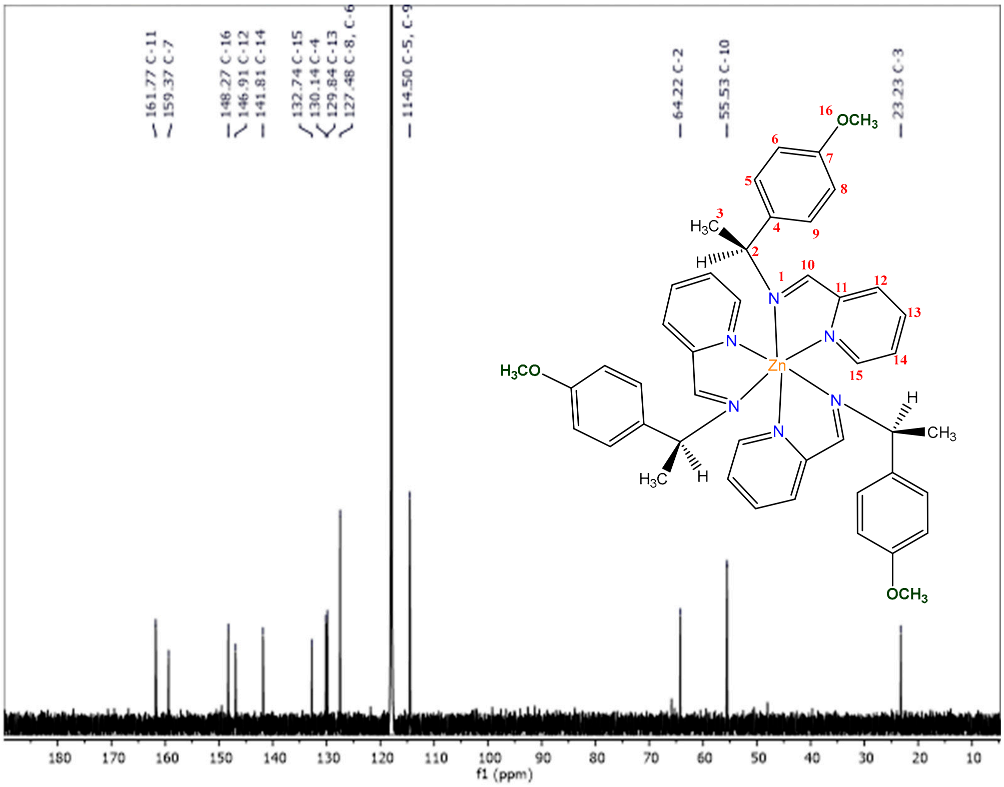

Figure S38. ^{13}C-NMR (100 MHz, CD_3CN) spectrum of complex **D**

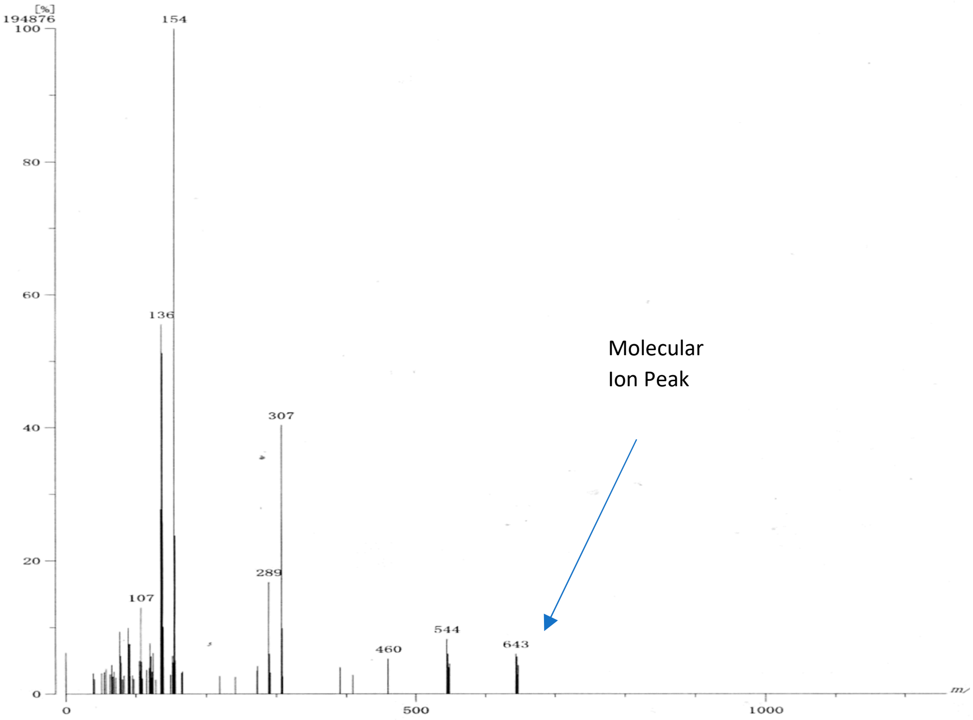

Figure S39. DART mass spectrum of complex **D** in the positive-ion mode.

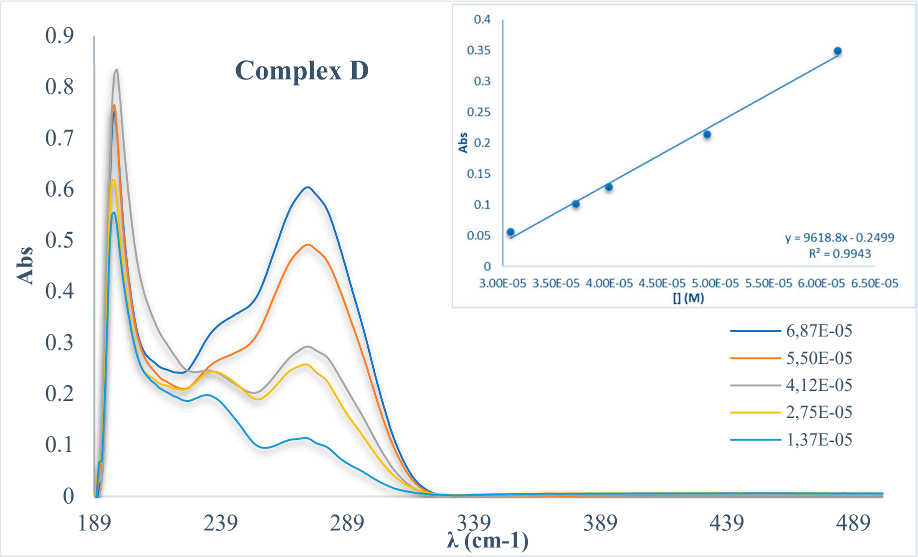

Figure S40. UV-Vis spectra (ACN) of complex **D**
