# Supplementary material for: Synthesis, Crystal Structures, Antimicrobial Activity, and Acute Toxicity Evaluation of Chiral Zn(II) Schiff Base Complexes
Source: Molecules. 2024 Nov 25;29(23):5555. doi: 10.3390/molecules29235555 (PMC11643661; doi:10.3390/molecules29235555)

checkCIF/PLATON report

Structure factors have been supplied for datablock(s) irznclo4_a_mo

THIS REPORT IS FOR GUIDANCE ONLY. IF USED AS PART OF A REVIEW PROCEDURE FOR PUBLICATION, IT SHOULD NOT REPLACE THE EXPERTISE OF AN EXPERIENCED CRYSTALLOGRAPHIC REFEREE.

No syntax errors found. CIF dictionary Interpreting this report

Datablock: irznclo4_a_mo

```
Bond precision:   C-C = 0.0096 A          Wavelength=0.71073

Cell:              a=19.2189(9)     b=12.9740(5)        c=18.6227(11)
                   alpha=90         beta=106.389(6)     gamma=90
Temperature:       293 K
```

	Calculated	Reported
Volume	4454.8(4)	4454.8(4)
Space group	C 2	C 1 2 1
Hall group	C 2y	C 2y
Moiety formula	C42 H39 F3 N6 Zn, 2(Cl O4) [+ solvent]	C42 H39 F3 N6 Zn, 2(Cl O4)
Sum formula	C42 H39 Cl2 F3 N6 O8 Zn [+ solvent]	C42 H39 Cl2 F3 N6 O8 Zn
Mr	949.08	949.06
Dx,g cm-3	1.415	1.415
Z	4	4
Mu (mm-1)	0.741	0.741
F000	1952.0	1952.0
F000'	1955.23	
h,k,lmax	25,17,24	25,17,24
Nref	10646[5555]	10605
Tmin,Tmax	0.802,0.886	0.843,0.956
Tmin'	0.802	

```
Correction method= # Reported T Limits: Tmin=0.843 Tmax=0.956
AbsCorr = GAUSSIAN

Data completeness= 1.91/1.00       Theta(max)= 27.873
```

R(reflections)= 0.0463(8530)   wR2(reflections)= 0.1294(10605)

S = 1.027   Npar= 563

The following ALERTS were generated. Each ALERT has the format
test-name_ALERT_alert-type_alert-level.
Click on the hyperlinks for more details of the test.

Alert level C

```
PLAT241_ALERT_2_C High   'MainMol' Ueq as Compared to Neighbors of         C1 Check
PLAT241_ALERT_2_C High   'MainMol' Ueq as Compared to Neighbors of         C2 Check
PLAT241_ALERT_2_C High   'MainMol' Ueq as Compared to Neighbors of         C7 Check
PLAT242_ALERT_2_C Low    'MainMol' Ueq as Compared to Neighbors of         C3 Check
PLAT242_ALERT_2_C Low    'MainMol' Ueq as Compared to Neighbors of         C4 Check
PLAT260_ALERT_2_C Large Average Ueq of Residue Including       Cl1      0.270 Check
PLAT260_ALERT_2_C Large Average Ueq of Residue Including       Cl2      0.172 Check
PLAT260_ALERT_2_C Large Average Ueq of Residue Including       Cl3      0.199 Check
PLAT334_ALERT_2_C Small <C-C> Benzene Dist.   C1       -C7       .       1.36 Ang.
PLAT341_ALERT_3_C Low Bond Precision on  C-C Bonds ..............    0.00956 Ang.
PLAT910_ALERT_3_C Missing # of FCF Reflection(s) Below Theta(Min).         10 Note
                1  1  0,   2  0  0,  -2  0  1,  -1  1  1,   0  0  1,   1  1  1,
                2  0  1,  -2  0  2,  -1  1  2,   0  0  2,
```

Alert level G

```
PLAT003_ALERT_2_G Number of Uiso or U(i,j) Restrained non-H Atoms           6 Report
PLAT177_ALERT_4_G The CIF-Embedded .res File Contains DELU Records          1 Report
PLAT178_ALERT_4_G The CIF-Embedded .res File Contains SIMU Records          1 Report
PLAT199_ALERT_1_G Reported _cell_measurement_temperature ..... (K)        293 Check
PLAT200_ALERT_1_G Reported   _diffrn_ambient_temperature ..... (K)        293 Check
PLAT244_ALERT_4_G Low    'Solvent' Ueq as Compared to Neighbors of        Cl1 Check
PLAT244_ALERT_4_G Low    'Solvent' Ueq as Compared to Neighbors of        Cl2 Check
PLAT244_ALERT_4_G Low    'Solvent' Ueq as Compared to Neighbors of        Cl3 Check
PLAT605_ALERT_4_G Largest Solvent Accessible VOID in the Structure        130 A**3
PLAT791_ALERT_4_G Model has Chirality at C5         (Sohncke SpGr)          R Verify
PLAT791_ALERT_4_G Model has Chirality at C21        (Sohncke SpGr)          R Verify
PLAT791_ALERT_4_G Model has Chirality at C35        (Sohncke SpGr)          R Verify
PLAT794_ALERT_5_G Tentative Bond Valency for Zn1       (II)       .      1.71 Info
PLAT860_ALERT_3_G Number of Least-Squares Restraints .............         49 Note
PLAT868_ALERT_4_G ALERTS Due to the Use of _smtbx_masks Suppressed          ! Info
PLAT933_ALERT_2_G Number of HKL-OMIT Records in Embedded .res File          2 Note
               -3 -1  1,  -3  1  1,
PLAT969_ALERT_5_G The 'Henn et al.' R-Factor-gap value ..........       4.441 Note
              Predicted wR2: Based on SigI**2  2.91 or SHELX Weight 12.60
PLAT978_ALERT_2_G Number C-C Bonds with Positive Residual Density.          0 Info
```

---

0 **ALERT level A** = Most likely a serious problem - resolve or explain
0 **ALERT level B** = A potentially serious problem, consider carefully
11 **ALERT level C** = Check. Ensure it is not caused by an omission or oversight
18 **ALERT level G** = General information/check it is not something unexpected

2 ALERT type 1 CIF construction/syntax error, inconsistent or missing data

```
12 ALERT type 2 Indicator that the structure model may be wrong or deficient
 3 ALERT type 3 Indicator that the structure quality may be low
10 ALERT type 4 Improvement, methodology, query or suggestion
 2 ALERT type 5 Informative message, check
```

It is advisable to attempt to resolve as many as possible of the alerts in all categories. Often the minor alerts point to easily fixed oversights, errors and omissions in your CIF or refinement strategy, so attention to these fine details can be worthwhile. In order to resolve some of the more serious problems it may be necessary to carry out additional measurements or structure refinements. However, the purpose of your study may justify the reported deviations and the more serious of these should normally be commented upon in the discussion or experimental section of a paper or in the "special_details" fields of the CIF. checkCIF was carefully designed to identify outliers and unusual parameters, but every test has its limitations and alerts that are not important in a particular case may appear. Conversely, the absence of alerts does not guarantee there are no aspects of the results needing attention. It is up to the individual to critically assess their own results and, if necessary, seek expert advice.

Publication of your CIF in IUCr journals

A basic structural check has been run on your CIF. These basic checks will be run on all CIFs submitted for publication in IUCr journals (*Acta Crystallographica*, *Journal of Applied Crystallography*, *Journal of Synchrotron Radiation*); however, if you intend to submit to *Acta Crystallographica Section C* or *E* or *IUCrData*, you should make sure that full publication checks are run on the final version of your CIF prior to submission.

Publication of your CIF in other journals

Please refer to the *Notes for Authors* of the relevant journal for any special instructions relating to CIF submission.

PLATON version of 15/07/2024; check.def file version of 15/07/2024

Datablock irznclo4_a_mo - ellipsoid plot

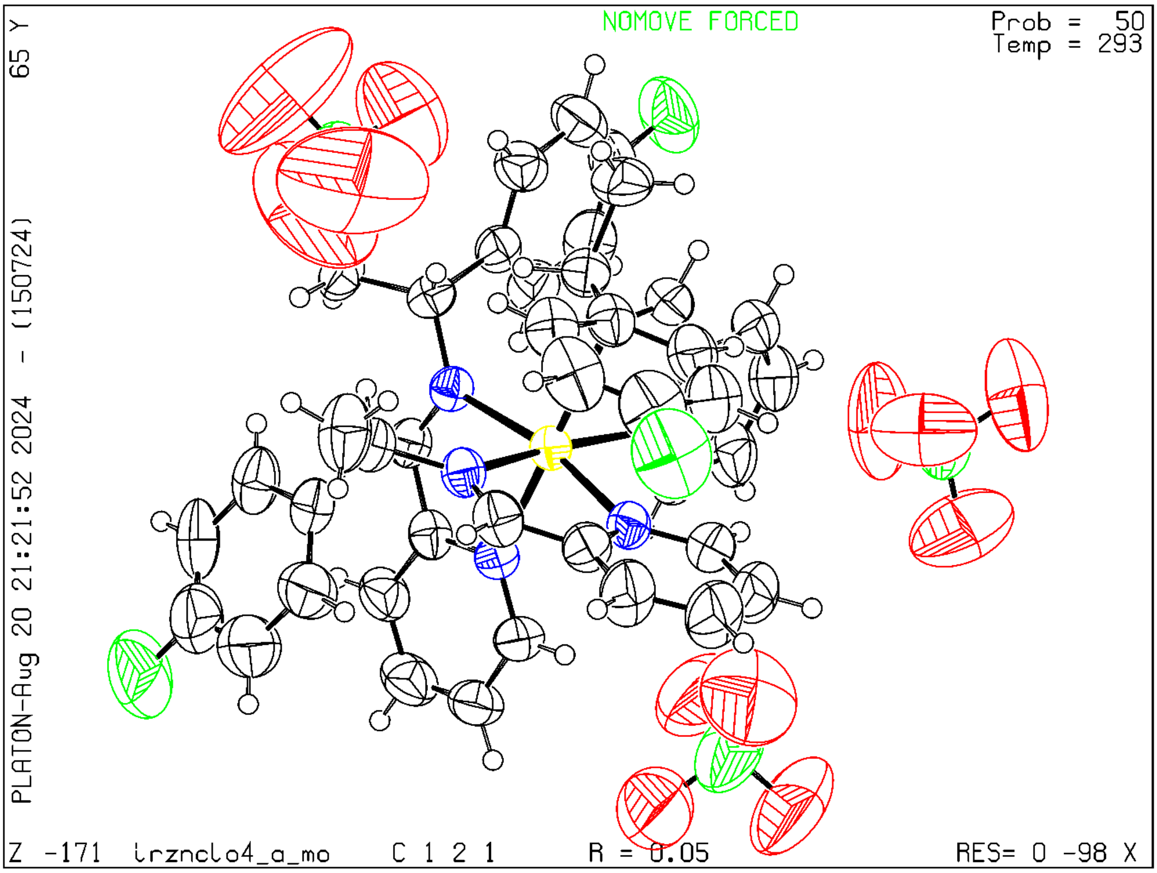

Supplement: Supplementary file 1 [file molecules-29-05555-s001.zip › complejo A.zip!/IRZncLO4_A_Mo.pdf]
