# Supplementary material for: Synthesis, Crystal Structures, Antimicrobial Activity, and Acute Toxicity Evaluation of Chiral Zn(II) Schiff Base Complexes
Source: Molecules. 2024 Nov 25;29(23):5555. doi: 10.3390/molecules29235555 (PMC11643661; doi:10.3390/molecules29235555)

checkCIF/PLATON report

You have not supplied any structure factors. As a result the full set of tests cannot be run.

THIS REPORT IS FOR GUIDANCE ONLY. IF USED AS PART OF A REVIEW PROCEDURE FOR PUBLICATION, IT SHOULD NOT REPLACE THE EXPERTISE OF AN EXPERIENCED CRYSTALLOGRAPHIC REFEREE.

No syntax errors found. CIF dictionary Interpreting this report

Datablock: imibzn_mo

Bond precision: C-C = 0.0067 A Wavelength=0.71073

Cell:	a=16.9023(3)	b=16.9023(3)	c=16.9023(3)
	alpha=90	beta=90	gamma=90
Temperature:	293 K		

	Calculated	Reported
Volume	4828.8(3)	4828.8(2)
Space group	P 21 3	P 21 3
Hall group	P 2ac 2ab 3	P 2ac 2ab 3
Moiety formula	C45 H48 N6 Zn, 2(Cl O4), C2 H3 N	C45 H48 N6 Zn1, 2(Cl1 O4), 0.33(C6 H9 N3)
Sum formula	C47 H51 Cl2 N7 O8 Zn	C47 H51 Cl2 N7 O8 Zn
Mr	978.24	978.22
Dx,g cm-3	1.346	1.346
Z	4	4
Mu (mm-1)	0.678	0.678
F000	2040.0	2040.0
F000’	2043.08	
h,k,lmax	21,21,21	21,20,21
Nref	3557[1958]	3552
Tmin,Tmax	0.792,0.855	0.947,0.972
Tmin’	0.720	

Correction method= # Reported T Limits: Tmin=0.947 Tmax=0.972
AbsCorr = ANALYTICAL

Data completeness= 1.81/1.00 Theta(max)= 27.085

R(reflections)= 0.0367(3004) wR2(reflections)= 0.1026(3552)

S = 1.049 Npar= 199

The following ALERTS were generated. Each ALERT has the format
test-name_ALERT_alert-type_alert-level.
Click on the hyperlinks for more details of the test.

Alert level C

```
PLAT042_ALERT_1_C Calc. and Reported MoietyFormula Strings  Differ     Please Check
              Calc: C45 H48 N6 Zn, 2(Cl O4), C2 H3 N
              Rep.: C45 H48 N6 Zn1, 2(Cl1 O4), 0.33(C6 H9 N3)
PLAT260_ALERT_2_C Large Average Ueq of Residue Including      Cl1      0.219 Check
PLAT260_ALERT_2_C Large Average Ueq of Residue Including      Cl2      0.153 Check
PLAT341_ALERT_3_C Low Bond Precision on  C-C Bonds ...............    0.00667 Ang.
```

Alert level G

```
FORMU01_ALERT_1_G  There is a discrepancy between the atom counts in the
            _chemical_formula_sum and _chemical_formula_moiety. This is
            usually due to the moiety formula being in the wrong format.
            Atom count from _chemical_formula_sum:   C47 H51 Cl2 N7 O8 Zn1
            Atom count from _chemical_formula_moiety:C46.98 H50.97 Cl2 N6.99 O8 Zn
PLAT012_ALERT_1_G No       _shelx_res_checksum Found in CIF ......     Please Check
PLAT199_ALERT_1_G Reported _cell_measurement_temperature ..... (K)        293 Check
PLAT200_ALERT_1_G Reported   _diffrn_ambient_temperature ..... (K)        293 Check
PLAT244_ALERT_4_G Low    'Solvent' Ueq as Compared to Neighbors of        Cl1 Check
PLAT244_ALERT_4_G Low    'Solvent' Ueq as Compared to Neighbors of        Cl2 Check
PLAT300_ALERT_4_G Atom Site Occupancy of H17A       Constrained at     0.3333 Check
PLAT300_ALERT_4_G Atom Site Occupancy of H17B       Constrained at     0.3333 Check
PLAT300_ALERT_4_G Atom Site Occupancy of H17C       Constrained at     0.3333 Check
PLAT789_ALERT_4_G Atoms with Negative _atom_site_disorder_group  #          3 Check
PLAT791_ALERT_4_G Model has Chirality at C7         (Sohncke SpGr)          S Verify
PLAT794_ALERT_5_G Tentative Bond Valency for Zn1       (II)      .       1.74 Info
PLAT822_ALERT_4_G CIF-embedded .res Contains Negative PART Numbers          1 Check
PLAT883_ALERT_1_G No Info/Value for _atom_sites_solution_primary .     Please Do !
```

0 **ALERT level A** = Most likely a serious problem - resolve or explain
0 **ALERT level B** = A potentially serious problem, consider carefully
4 **ALERT level C** = Check. Ensure it is not caused by an omission or oversight
14 **ALERT level G** = General information/check it is not something unexpected

6 ALERT type 1 CIF construction/syntax error, inconsistent or missing data
2 ALERT type 2 Indicator that the structure model may be wrong or deficient
1 ALERT type 3 Indicator that the structure quality may be low
8 ALERT type 4 Improvement, methodology, query or suggestion
1 ALERT type 5 Informative message, check

It is advisable to attempt to resolve as many as possible of the alerts in all categories. Often the minor alerts point to easily fixed oversights, errors and omissions in your CIF or refinement strategy, so attention to these fine details can be worthwhile. In order to resolve some of the more serious problems it may be necessary to carry out additional measurements or structure refinements. However, the purpose of your study may justify the reported deviations and the more serious of these should normally be commented upon in the discussion or experimental section of a paper or in the "special_details" fields of the CIF. checkCIF was carefully designed to identify outliers and unusual parameters, but every test has its limitations and alerts that are not important in a particular case may appear. Conversely, the absence of alerts does not guarantee there are no aspects of the results needing attention. It is up to the individual to critically assess their own results and, if necessary, seek expert advice.

Publication of your CIF in IUCr journals

A basic structural check has been run on your CIF. These basic checks will be run on all CIFs submitted for publication in IUCr journals (*Acta Crystallographica*, *Journal of Applied Crystallography*, *Journal of Synchrotron Radiation*); however, if you intend to submit to *Acta Crystallographica Section C* or *E* or *IUCrData*, you should make sure that full publication checks are run on the final version of your CIF prior to submission.

Publication of your CIF in other journals

Please refer to the *Notes for Authors* of the relevant journal for any special instructions relating to CIF submission.

PLATON version of 15/07/2024; check.def file version of 15/07/2024

Datablock imibzn_mo - ellipsoid plot

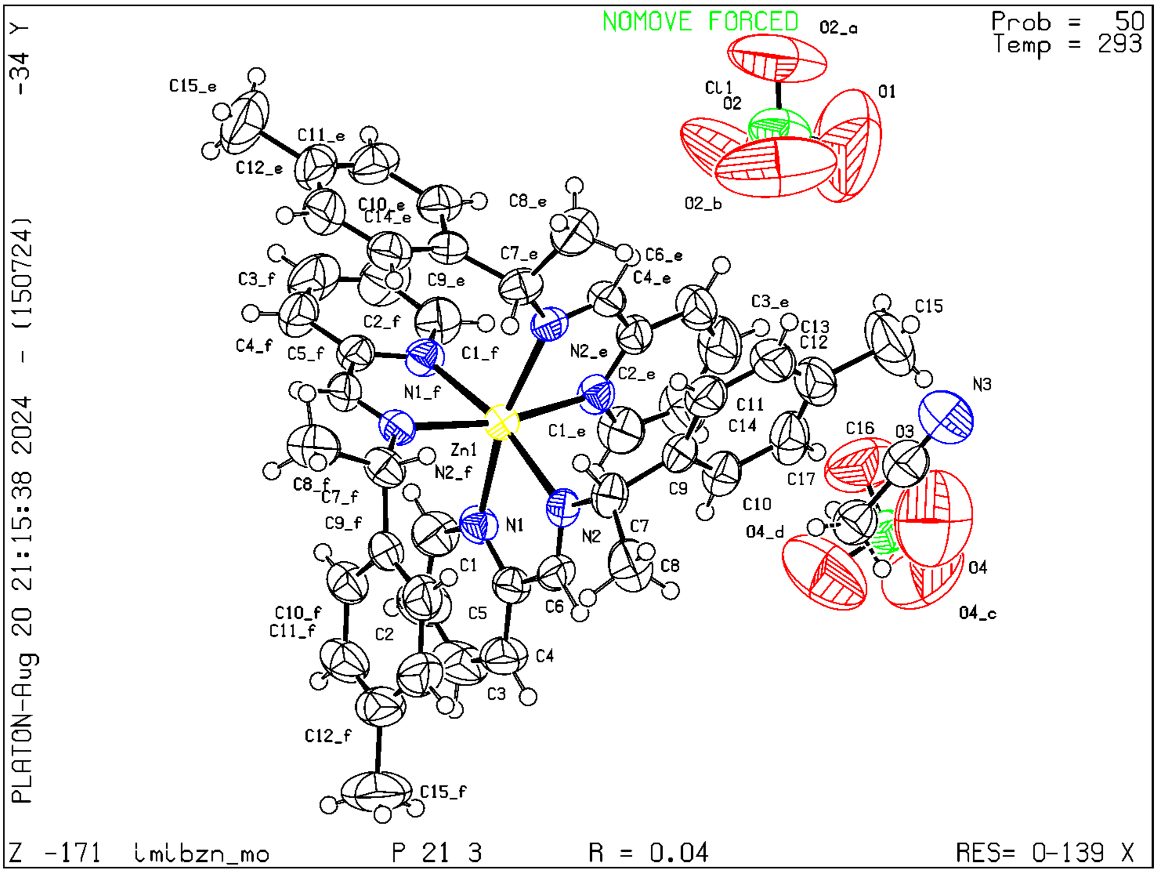

Supplement: Supplementary file 1 [file molecules-29-05555-s001.zip › complejo B.zip!/ImIBZn_Mo.pdf]
