# Supplementary material for: Synthesis, Crystal Structures, Antimicrobial Activity, and Acute Toxicity Evaluation of Chiral Zn(II) Schiff Base Complexes
Source: Molecules. 2024 Nov 25;29(23):5555. doi: 10.3390/molecules29235555 (PMC11643661; doi:10.3390/molecules29235555)

checkCIF/PLATON report

You have not supplied any structure factors. As a result the full set of tests cannot be run.

THIS REPORT IS FOR GUIDANCE ONLY. IF USED AS PART OF A REVIEW PROCEDURE FOR PUBLICATION, IT SHOULD NOT REPLACE THE EXPERTISE OF AN EXPERIENCED CRYSTALLOGRAPHIC REFEREE.

No syntax errors found. CIF dictionary Interpreting this report

Datablock: i-jzn_a_mo

Bond precision:	C-C = 0.0089 A		Wavelength=0.71073
Cell:	a=17.5430(3)	b=17.5430(3)	c=17.5430(3)
	alpha=90	beta=90	gamma=90
Temperature:	293 K		

	Calculated	Reported
Volume	5399.0(3)	5399.0(3)
Space group	P 21 3	P 21 3
Hall group	P 2ac 2ab 3	P 2ac 2ab 3
Moiety formula	C45 H48 N6 O3 Zn, 2(Cl O4), 3(C2 H3 N)	C45 H48 N6 O3 Zn1, 2(Cl1 O4), 3(C2 H3 N)
Sum formula	C51 H57 Cl2 N9 O11 Zn	C51 H57 Cl2 N9 O11 Zn
Mr	1108.35	1108.32
Dx,g cm-3	1.364	1.364
Z	4	4
Mu (mm-1)	0.620	0.620
F000	2312.0	2312.0
F000’	2315.25	
h,k,lmax	21,21,21	21,21,21
Nref	3671[2016]	3663
Tmin,Tmax	0.694,0.790	0.946,0.965
Tmin’	0.666	

Correction method= # Reported T Limits: Tmin=0.946 Tmax=0.965
AbsCorr = ANALYTICAL

Data completeness= 1.82/1.00 Theta(max)= 26.347

R(reflections)= 0.0461(2843) wR2(reflections)= 0.1351(3663)

S = 1.070 Npar= 226

The following ALERTS were generated. Each ALERT has the format
test-name_ALERT_alert-type_alert-level.
Click on the hyperlinks for more details of the test.

Alert level C

```
PLAT042_ALERT_1_C Calc. and Reported MoietyFormula Strings  Differ     Please Check
              Calc: C45 H48 N6 O3 Zn, 2(Cl O4), 3(C2 H3 N)
              Rep.: C45 H48 N6 O3 Zn1, 2(Cl1 O4), 3(C2 H3 N)
PLAT244_ALERT_4_C Low    'Solvent' Ueq as Compared to Neighbors of        C17 Check
PLAT260_ALERT_2_C Large Average Ueq of Residue Including       Cl1      0.278 Check
PLAT260_ALERT_2_C Large Average Ueq of Residue Including       Cl2      0.159 Check
PLAT260_ALERT_2_C Large Average Ueq of Residue Including        N3      0.177 Check
PLAT341_ALERT_3_C Low Bond Precision on  C-C Bonds ...............    0.00893 Ang.
```

Alert level G

```
PLAT012_ALERT_1_G No       _shelx_res_checksum Found in CIF ......     Please Check
PLAT063_ALERT_4_G Crystal Size Possibly too Large for Beam Size ..       0.64 mm
PLAT180_ALERT_4_G Check Cell Rounding: # of Values Ending with 0 =          3 Note
PLAT199_ALERT_1_G Reported _cell_measurement_temperature ..... (K)        293 Check
PLAT200_ALERT_1_G Reported   _diffrn_ambient_temperature ..... (K)        293 Check
PLAT244_ALERT_4_G Low    'Solvent' Ueq as Compared to Neighbors of        Cl1 Check
PLAT244_ALERT_4_G Low    'Solvent' Ueq as Compared to Neighbors of        Cl2 Check
PLAT791_ALERT_4_G Model has Chirality at C7         (Sohncke SpGr)          S Verify
PLAT794_ALERT_5_G Tentative Bond Valency for Zn1       (II)      .       1.70 Info
PLAT883_ALERT_1_G No Info/Value for _atom_sites_solution_primary .     Please Do !
```

```
   0 ALERT level A = Most likely a serious problem - resolve or explain
   0 ALERT level B = A potentially serious problem, consider carefully
   6 ALERT level C = Check. Ensure it is not caused by an omission or oversight
  10 ALERT level G = General information/check it is not something unexpected

   5 ALERT type 1 CIF construction/syntax error, inconsistent or missing data
   3 ALERT type 2 Indicator that the structure model may be wrong or deficient
   1 ALERT type 3 Indicator that the structure quality may be low
   6 ALERT type 4 Improvement, methodology, query or suggestion
   1 ALERT type 5 Informative message, check
```

It is advisable to attempt to resolve as many as possible of the alerts in all categories. Often the minor alerts point to easily fixed oversights, errors and omissions in your CIF or refinement strategy, so attention to these fine details can be worthwhile. In order to resolve some of the more serious problems it may be necessary to carry out additional measurements or structure refinements. However, the purpose of your study may justify the reported deviations and the more serious of these should normally be commented upon in the discussion or experimental section of a paper or in the "special_details" fields of the CIF. checkCIF was carefully designed to identify outliers and unusual parameters, but every test has its limitations and alerts that are not important in a particular case may appear. Conversely, the absence of alerts does not guarantee there are no aspects of the results needing attention. It is up to the individual to critically assess their own results and, if necessary, seek expert advice.

Publication of your CIF in IUCr journals

A basic structural check has been run on your CIF. These basic checks will be run on all CIFs submitted for publication in IUCr journals (*Acta Crystallographica*, *Journal of Applied Crystallography*, *Journal of Synchrotron Radiation*); however, if you intend to submit to *Acta Crystallographica Section C* or *E* or *IUCrData*, you should make sure that full publication checks are run on the final version of your CIF prior to submission.

Publication of your CIF in other journals

Please refer to the *Notes for Authors* of the relevant journal for any special instructions relating to CIF submission.

PLATON version of 15/07/2024; check.def file version of 15/07/2024

Datablock i-jzn_a_mo - ellipsoid plot

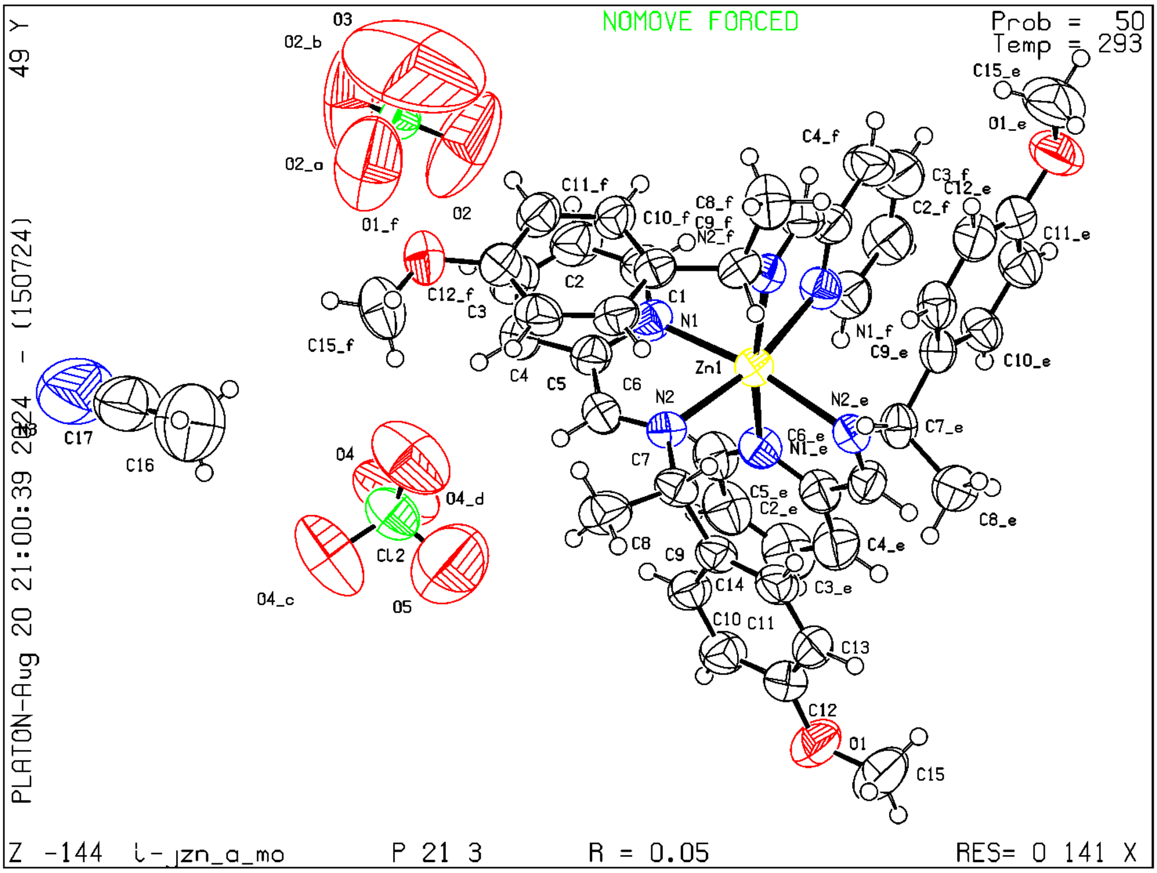

Supplement: Supplementary file 1 [file molecules-29-05555-s001.zip › complejo D.zip!/I-J+Zn_a_Mo.pdf]
